# Amniotic-fluid metabolomics identifies phospholipid remodeling as a metabolic signature of intrauterine exposure in pregnancies with polycystic ovary syndrome

**DOI:** 10.3389/fendo.2026.1904695

**Published:** 2026-09-04

**Authors:** Shimin Wu, Jiayu Shi, Chenyan He, Jie Chen, Qian Guo, Shuangyi Li, Junhua Tong, Qianyu Chen, Ziyi Yan, Ji Du, Xiaoyu Yu, Kaixuan Lin, Yong Tan

**Affiliations:** 1 Affiliated Hospital of Nanjing University of Chinese Medicine, Nanjing, China; 2 Nanjing University of Chinese Medicine, Nanjing, China; 3 Suzhou Hospital of Traditional Chinese Medicine, Suzhou, China

**Keywords:** amniotic fluid, intrauterine exposure, metabolomics, phospholipid remodeling, placenta, polycystic ovary syndrome, pregnancy

## Abstract

**Background:**

Polycystic ovary syndrome is associated with metabolic and hormonal disturbances during pregnancy, but whether these alterations are reflected in the fetal intrauterine exposure environment remains incompletely understood. This study aimed to identify polycystic ovary syndrome-related intrauterine metabolic signatures using late-gestation amniotic fluid.

**Methods:**

Untargeted metabolomic profiling was performed on late-gestation amniotic fluid samples from women with polycystic ovary syndrome and controls. Differential metabolite analysis, pathway-level analysis, and multilevel sensitivity analyses were conducted to identify robust metabolic alterations associated with maternal polycystic ovary syndrome. Exploratory placental transcriptomic analysis and targeted RT-qPCR assessment in an independent sample set were further used to examine tissue-level molecular changes related to the lipidomic findings.

**Results:**

Amniotic fluid from pregnancies with polycystic ovary syndrome showed a distinct metabolic profile dominated by lipid remodeling. Differential metabolites were mainly enriched in membrane phospholipids, sphingolipid-related metabolites, polyunsaturated fatty acid-related pathways, and selected steroid hormone-related metabolites. Phospholipid remodeling was characterized by decreased phosphatidylcholine species, increased phosphatidylethanolamine species, a lower phosphatidylcholine/phosphatidylethanolamine ratio, and redistribution of arachidonic acid-containing phospholipids. Alpha-linolenic acid metabolism provided an additional polyunsaturated fatty acid-related signal. Sphingolipid enrichment suggested that lipid alterations extended from membrane structural remodeling to lipid-mediated signaling. Selected steroid hormone-related metabolites were also elevated, consistent with an altered hormonal milieu in pregnancies with polycystic ovary syndrome. These signatures remained largely stable across sensitivity analyses, as did the multivariable-adjusted inverse association between PE(16:0/20:4) and birth weight. Exploratory placental transcriptomic analysis and targeted RT-qPCR assessment in a small independent cohort provided preliminary tissue-level support for phospholipid-related molecular alterations, with the clearest changes involving PLA2-family genes and PCYT1A, suggesting cross-cohort convergence at the pathway level.

**Conclusions:**

These findings identify phospholipid remodeling as a major amniotic-fluid metabolic signature of polycystic ovary syndrome-related intrauterine exposure. PUFA-related metabolism, sphingolipid enrichment, and steroid hormone-related alterations provide additional metabolic context. Complementary placental molecular findings and the inverse association between PE(16:0/20:4) and birth weight further suggest potential links with the maternal–fetal interface and fetal growth. Further studies are needed to clarify the biological relevance of these changes and their potential role in offspring metabolic susceptibility.

## 1 Introduction

Polycystic ovary syndrome(PCOS), recently proposed to be renamed polyendocrine metabolic ovarian syndrome(PMOS) (1), is a common but complex endocrine metabolic disorder that affects women throughout their entire life cycle (2, 3). It is manifested as irregular menstruation and hyperandrogenemia in adolescence, infertility and pregnancy complications during gestation (4, 5), and metabolic irregularities and endometrial malignancies in post-menopause. In addition, the offspring of women with PCOS are not only at a significantly higher risk of preterm birth, congenital anomalies, cardiovascular and urogenital defects, as well as metabolic disorders, their daughters are also more likely to develop PCOS themselves, with some large-scale cohort studies even suggesting an approximately five-fold increase in risk (5, 6). Accumulating evidence suggests that maternal endocrine and metabolic abnormalities associated with PCOS may increase the health risks of offspring by altering the intrauterine environment (7–10). Such changes in the intrauterine environment may further exacerbate genetic susceptibility through interactions between the mother and the fetus, thereby increasing the risk of developing PCOS later in life (11–15). Rather than a single factor, this is most likely the outcome of a continuous interaction between genetics, hormones, and the environment.

Nevertheless, the mechanism of its maternal-fetal transmission is not yet completely understood. Previous studies have explored the impact of PCOS on offspring health from the perspectives of high intrauterine androgen and anti-Müllerian hormone exposure (14, 16–18), suggesting that these endocrine and metabolic factors may be involved in the formation of disease susceptibility in early fetal development (19). However, the majority of current research relies on maternal serum, placental, and neonatal umbilical artery blood, and implies from their respective phenotypes. This approach limits the direct characterization of the fetus’s actual intrauterine metabolic environment.

As is known to all, the fetus is directly exposed to amniotic fluid during pregnancy, which more accurately represents the fetus’s actual intrauterine exposure environment. During pregnancy, the fetus exchanges substances with amniotic fluid, which is composed of metabolites that incorporate metabolic information from the mother, placenta, and fetus. These metabolites include amino acids, carbohydrates, organic acids, lipids, steroid hormones, and other small-molecule metabolites. Thus, it would be more helpful in directly determining the fetus’s intrauterine metabolic and endocrine status in comparison to other biological samples. Moreover, as pregnancy progresses into the third trimester, fetal metabolic activity increases, accompanied by ongoing endocrine and metabolic alterations (20). The metabolic abnormalities may accumulate and become more apparent. The intrauterine environment offers a more precise representation of the fetus’s current physiological state, thereby providing an opportunity to capture metabolic traits associated with PCOS.

Metabolomics is the discipline that qualitatively and quantitatively analyzes all low molecular weight metabolites in organisms or cells to disclose their relationship with physiological and pathological changes. Amniotic fluid metabolomics has been shown to reflect fetal disease risk before birth and predict neonatal and offspring health risks (21–25). Therefore, analyzing the intrauterine environmental changes in PCOS-related pregnancies from an amniotic fluid metabolomics perspective, can help reveal the potential pathways by which maternal PCOS-related endocrine and metabolic disorders affect the fetal developmental environment.

Therefore, to determine if PCOS-related intrauterine environmental alterations are marked by metabolic abnormalities and whether these abnormalities correlate with fetal developmental outcomes, this study utilized amniotic fluid from late pregnancy, compared the non-targeted metabolomics characteristics of pregnant women with PCOS to those of healthy pregnant women. Additionally, we performed differential metabolite screening, pathway enrichment, lipid and steroid metabolism analysis, sensitivity analysis, and clinical outcome correlation analysis to systematically assess PCOS-related intrauterine metabolic environmental changes. This exploratory analysis will be used as a foundation for subsequent targeted validation of the identified metabolites, animal experiments, and mechanistic studies that are supported by these discoveries. By linking amniotic fluid metabolic changes with PCOS-related intrauterine exposure, this approach offers a unique method of understanding of maternal–fetal metabolic transmission in PCOS, providing evidence for addressing offspring disease susceptibility in clinical practice.

## 2 Materials and methods

### 2.1 Study participants

Pregnant women undergoing cesarean delivery were recruited from the Department of Obstetrics and Reproductive Medicine at Jiangsu Province Hospital of Chinese Medicine (Zidong Campus) between September 2023 and December 2025. A total of 50 participants were enrolled, including 25 women with polycystic ovary syndrome (PCOS) and 25 controls. PCOS was diagnosed according to the Rotterdam criteria. Control participants had regular menstrual cycles (21–35 days) and normal endocrine profiles. All participants had singleton or twin pregnancies at 36–41 weeks of gestation.

Participants aged 25–36 years were eligible for inclusion. Women with pre-existing diabetes or hypertension, other major endocrine disorders that could substantially affect metabolic status, severe hepatic or renal dysfunction, autoimmune disease, active infection, fetal abnormalities, or incomplete clinical data were excluded. After quality control and follow–up, 22 women with PCOS and 23 controls were included in the final analysis (**Supplementary Table 1**).

### 2.2 Ethics approval

The study was approved by the Ethics Committee of Jiangsu Province Hospital of Chinese Medicine (Approval No. 2023NL–084–03). All participants provided written informed consent, and all procedures were conducted in accordance with the Declaration of Helsinki.

### 2.3 Amniotic fluid collection

Amniotic fluid samples were obtained during elective cesarean delivery in late gestation. Prior to fetal delivery, fluid was aspirated under sterile conditions from the uterine cavity, avoiding the placental attachment site. The initial 1–2 mL was discarded to minimize potential maternal blood contamination, and 5–10 mL of amniotic fluid was subsequently collected. Samples were immediately placed on ice and processed within 30 minutes. After centrifugation at 4°C (1500 rpm, 10 min), supernatants were separated, aliquoted, and stored at –80°C until further analysis. All procedures were performed under sterile conditions.

### 2.4 Metabolite extraction

Samples were thawed at 4°C and extracted with pre–chilled methanol (1:3, v/v), followed by incubation at –20°C for 30 min. After centrifugation at 4°C (2,300 g, 20 min), supernatants were collected for Liquid chromatography-tandem mass spectrometry(LC–MS/MS) analysis.

### 2.5 LC–MS/MS analysis

LC–MS/MS analysis was performed using a UHPLC system (Shimadzu, Japan) coupled to a Q Exactive Plus mass spectrometer (Thermo Scientific, USA). Chromatographic separation was achieved on an ACQUITY UPLC HSS T3 column (2.1×100 mm, 1.8 ųm; Waters, USA) with 0.1% formic acid in water and acetonitrile as the mobile phases. A linear gradient from 2% to 100% acetonitrile was applied over 13 min at a flow rate of 0.3 mL/min, with the column temperature maintained at 40 °C. Data were acquired in both positive and negative ion modes over an m/z range of 75–1050, followed by data-dependent MS/MS acquisition of the top 10 precursor ions using stepped higher-energy collisional dissociation energies of 20, 30, and 40 eV. Detailed acquisition parameters are provided in **Supplementary Table 2**.

### 2.6 Data preprocessing

**Figure 1:**
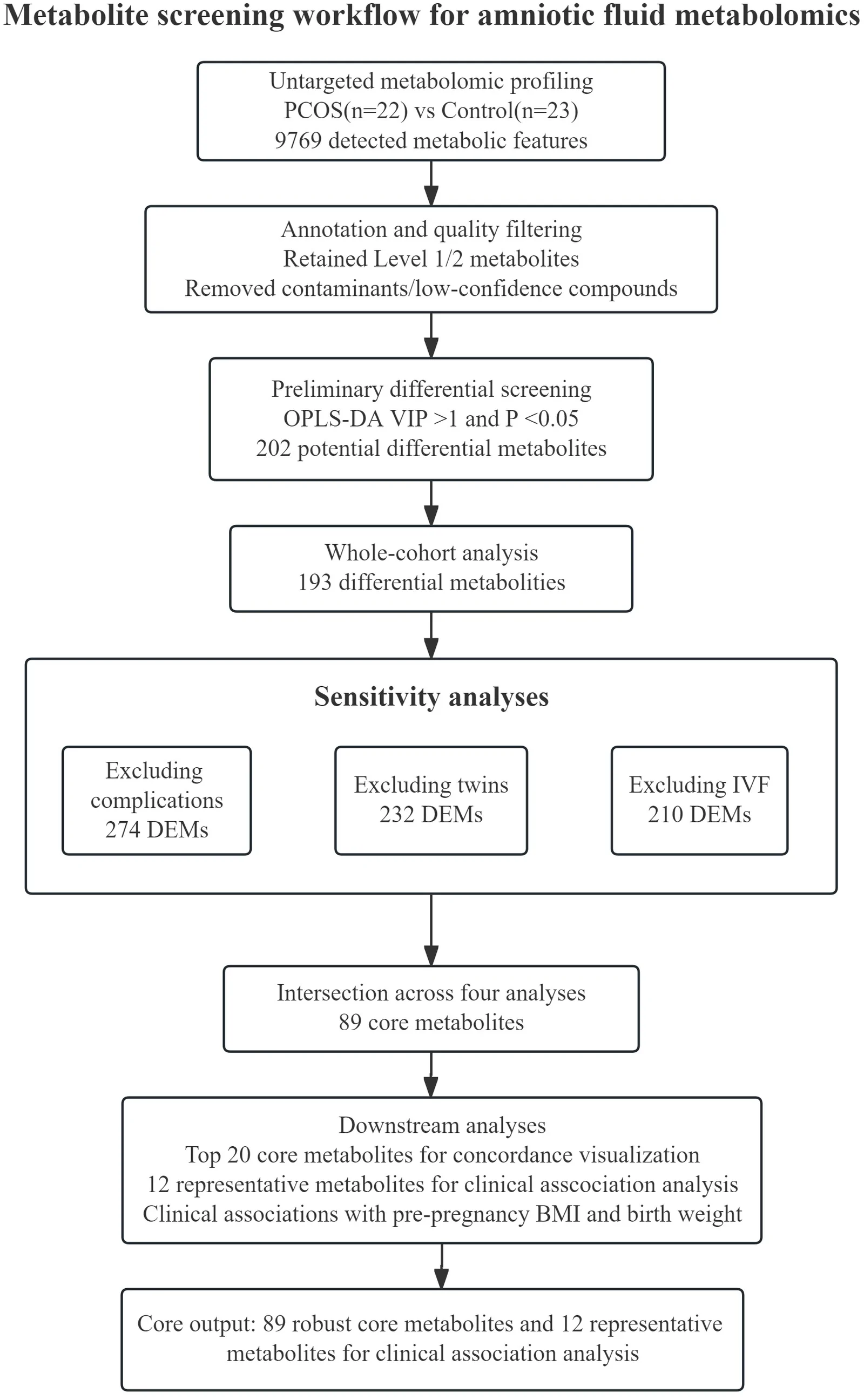
Workflow of amniotic-fluid metabolite selection and preprocessing. A total of 9,769 metabolic features were detected by untargeted amniotic-fluid metabolomics. Initial filtering retained Level 1/2 annotated metabolites and removed exogenous, low-confidence, and contaminant features. Differential metabolites were screened using OPLS-DA VIP values and uncorrected P values. Sensitivity analyses were then performed by excluding pregnancies with complications, twin gestations, or IVF conception. Metabolites consistently retained across the whole-cohort and sensitivity analyses were defined as the final core set.

Raw LC–MS data were processed using MS-DIAL for peak detection, alignment, and metabolite annotation against HMDB, MassBank, GNPS, and an in-house library. Features with more than 50% missing values were removed, and the remaining data were normalized to total ion intensity. The metabolite selection and preprocessing workflow is shown in **Figure 1**.

### 2.7 Placental RNA sequencing

Placental tissue samples from 8 women with PCOS and 6 controls were used for RNA sequencing (RNA-seq). Placental tissues were collected immediately after delivery from the middle section approximately 5 cm from the umbilical cord insertion site, avoiding areas of infarction or calcification. Visible blood contamination was gently removed, and tissues were blotted dry, placed in RNAlater at 4 °C overnight, and then stored at −80 °C until RNA extraction.

Total RNA was extracted using TRIzol reagent (Invitrogen, USA). RNA quality was assessed using a NanoDrop ND-1000 spectrophotometer, an Agilent Bioanalyzer 2100 system, and agarose gel electrophoresis. Samples meeting quality criteria were used for poly(A)-enriched cDNA library construction. Paired-end sequencing was performed on an Illumina NovaSeq 6000 platform using a PE150 strategy by LC-Bio Technology Co., Ltd. (Hangzhou, China).

### 2.8 Bioinformatics analysis of placental RNA-seq data

Raw reads were filtered using fastp and aligned to the human reference genome GRCh38 using HISAT2. Transcript assembly was performed using StringTie, with merged annotations generated by gffcompare. Gene expression levels were normalized as fragments per kilobase of transcript per million mapped reads for descriptive visualization. Differential expression between the PCOS and control groups was analyzed using edgeR. Fold changes (FC) were expressed as PCOS relative to control, so that positive log2 fold changes indicate higher expression in PCOS placentas. Differentially expressed genes were defined as those with P < 0.05 and FC ≥ 1.5 or ≤ 0.667 (|log2FC| ≥ log2(1.5) ≈ 0.585).

Principal component analysis was performed on log2(expression + 1) values of the 500 most variable genes, and sample-to-sample relationships were summarized by pairwise Pearson correlation with unsupervised hierarchical clustering.

Gene Ontology (GO) and Kyoto Encyclopedia of Genes and Genomes (KEGG) enrichment analyses were performed using DAVID, separately for all differentially expressed genes and for the upregulated and downregulated subsets. P values were adjusted using the Benjamini–Hochberg procedure, and terms with a false discovery rate (FDR) < 0.05 were considered significantly enriched.

### 2.9 RT-qPCR

RT-qPCR was performed using placental samples from an independent cohort of 4 women with PCOS and 4 controls, none of whom were included in the RNA-seq cohort. Tissues were collected from the same placental region described in Section 2.7 and stored directly at −80 °C.

Total RNA was extracted using the FastPure Complex Tissue/Cell Total RNA Isolation Kit (Vazyme Biotech, China), and RNA concentration was measured with a NanoDrop 2000 spectrophotometer (Thermo Fisher Scientific, USA). One microgram of RNA was reverse-transcribed using the First-Strand cDNA Synthesis Kit (Vazyme Biotech, China). The resulting cDNA was diluted tenfold before RT-qPCR. Reactions were carried out in a final volume of 20 μL, with an initial denaturation at 95 °C for 5 min, followed by 40 cycles at 95 °C for 15 s and 60 °C for 34 s. Each sample was analyzed in technical triplicate. ACTB and TBP were used as reference genes based on their reported stability in PCOS-related and human placental tissues (26, 27), respectively. Consistent with these reports, neither ACTB nor TBP showed a significant difference between the PCOS and control groups in our RNA-seq dataset. Relative expression was normalized to the geometric mean of ACTB and TBP and calculated using the 2^−ΔΔCt^ method. Statistical comparisons between groups were performed using normalized ΔCt values with Mann–Whitney U test. The clinical characteristics of the RT-qPCR cohort and the primer sequences are provided in **Supplementary Tables 9** and **S10**, respectively.

### 2.10 Statistical analysis

All statistical analyses were performed using R (version 4.5.2), MetaboAnalyst (https://www.metaboanalyst.ca/), and GraphPad Prism(version 10.1.2). Continuous variables were compared using Student’s t–test or Mann–Whitney U test, as appropriate, whereas categorical variables were compared using Chi–square or Fisher’s exact test. All tests were two–sided, and P < 0.05 was considered statistically significant. Omics data were normalized as described in the corresponding methodological sections before downstream analyses. Quality control (QC) sample consistency was evaluated using Pearson correlation, with r > 0.9 indicating high reproducibility, and Hotelling’s T² test with 95% or 99% confidence intervals was used to identify potential outliers. Where multiple comparisons were performed, significance was adjusted using false discovery rate (FDR) correction to control for type I error.

## 3 Results

### 3.1 Baseline characteristics

**Table 1** summarizes clinical baseline data, including age, pre-pregnancy BMI, BMI at delivery, gravidity, and parity, presented as mean ± SD. No significant differences were observed between the two groups in age, BMI, gravidity, parity, gestational age at delivery, or fetal sex (**Table 1**). However, neonates in the PCOS group had significantly lower birth weight than controls. The PCOS group also had a higher proportion of multiple pregnancies and a different mode of conception, with lower rates of natural conception and higher rates of ovulation induction and *in vitro* fertilization(IVF-ET). Detailed clinical information is provided in **Supplementary Table 1**.

**Table 1:** Baseline clinical characteristics of the study population.

Clinical variables	Control	PCOS	P value
Age	29.17 ± 3.157	30.00 ± 3.409	0.3734
Pre-pregnancy BMI	22.35 ± 3.3791	23.78 ± 3.8906	0.1943
BMI at delivery	26.74 ± 3.512	27.78 ± 4.327	0.3833
Gravidity	1.609 ± 0.9409	1.455 ± 0.8004	0.603
Parity	1.217 ± 0.4217	1.091 ± 0.2942	0.414
GA Delivery	38.90 ± 0.9506	38.67 ± 1.425	0.6561
Birth Weight	3580 ± 463.7	3057 ± 532.7	0.0012*
Plurality			
Singleton	23 (100%)	18 (81.82%)	0.0491*
Twin-DCDA	0 (0%)	2 (9.09%)	
Twin-MCDA	0 (0%)	2 (9.09%)	
Conception			
Spontaneous	23 (100%)	13 (59.09%)	0.0028*
Ovulation Induction	0 (0%)	6 (27.27%)	
IVF	0 (0%)	3 (13.64%)	
Fetal Sex			
Male	15 (65.22%)	8 (36.36%)	0.1392
Female	8 (34.78%)	14 (63.64%)	

There were no significant differences between the two groups in age, pre-pregnancy BMI, BMI at delivery, gravidity, parity, gestational age at delivery, or fetal sex (all P > 0.05). The PCOS group had a significantly lower birth weight, a higher proportion of multiple pregnancies, including dichorionic diamniotic (DCDA) and monochorionic diamniotic (MCDA) twins,and a higher frequency of assisted conception, including ovulation induction and *in vitro* fertilization (IVF), compared with controls (all P < 0.05). *P < 0.05.

### 3.2 Sample metabolomic analysis

Quality control samples clustered tightly in both ion modes, with Pearson correlation coefficients greater than 0.9 and no outliers by Hotelling’s T² test, confirming stable acquisition and good data reproducibility (**Supplementary Figures 1**–**3**). OPLS-DA showed separation between polycystic ovary syndrome and control amniotic-fluid profiles, with acceptable model performance (Q²> 0.5; **Supplementary Figure 4**).

### 3.3 Metabolite selection and data quality control

After preprocessing and metabolite selection, a set of high-confidence annotated metabolites was retained for downstream analysis. The workflow included feature detection, metabolite annotation, quality filtering, contaminant removal, missing-value filtering, and normalization, as summarized in **Figure 1**. These curated metabolites were then used for multivariate analysis, differential metabolite screening, pathway enrichment, and correlation analyses.

### 3.4 Global and single–variable analysis of differential metabolites

**Figure 2:**
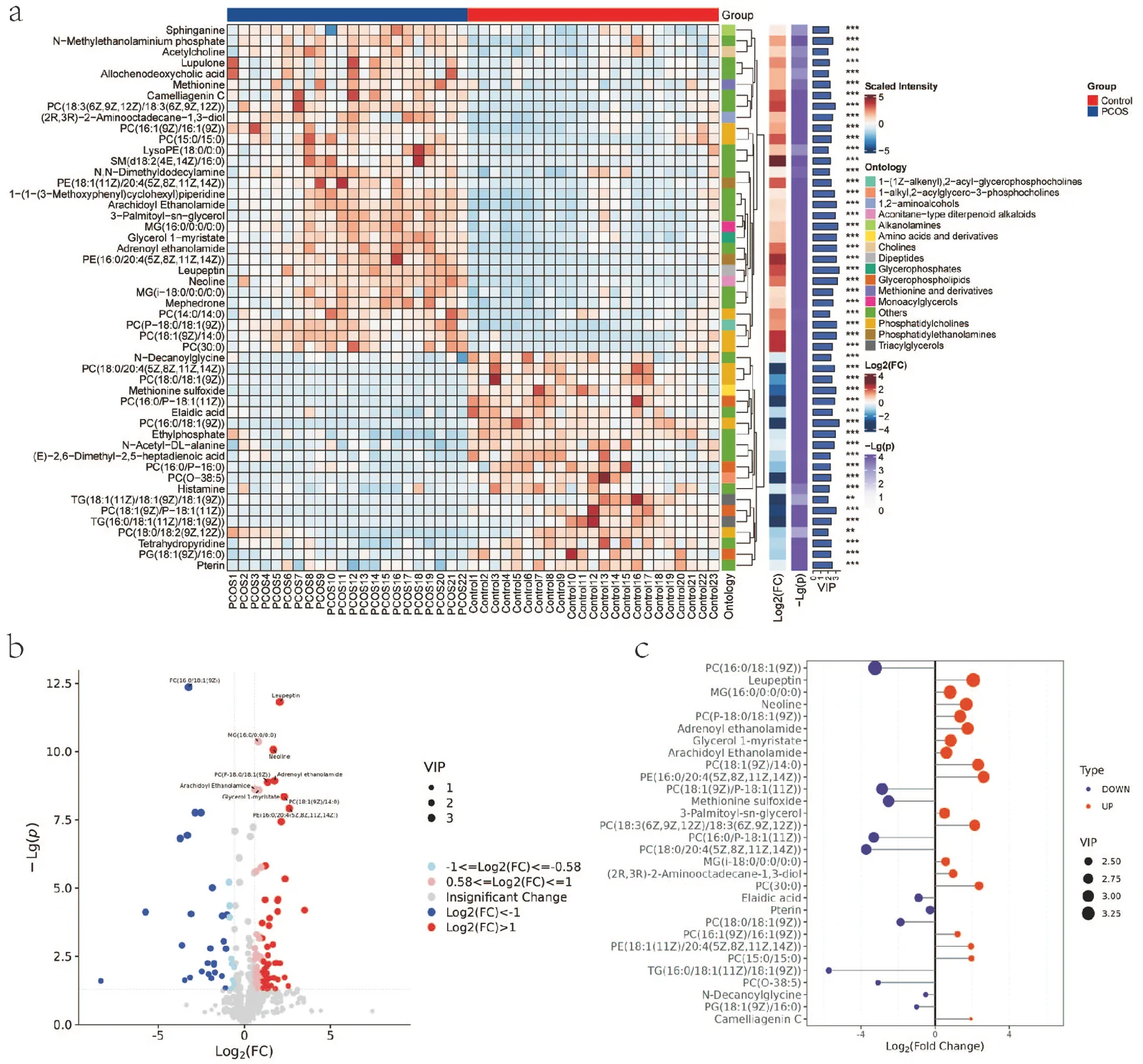
Differential metabolite profiles in amniotic fluid from pregnancies with polycystic ovary syndrome and controls. **(a)** Hierarchical clustering heatmap of the top 50 candidate differential metabolites ranked by OPLS-DA VIP scores. Rows represent metabolites, and columns represent samples. Z-score-normalized abundance is shown by color. Side annotations indicate metabolite class, log2 fold change, −log10 P value, and VIP score. **(b)** Volcano plot of differential metabolites between the two groups. Each point represents one metabolite. Differential metabolites were defined as fold change ≥1.5 or ≤1/1.5 with P < 0.05. A total of 138 metabolites were upregulated and 64 were downregulated in polycystic ovary syndrome amniotic fluid. **(c)** Top 30 candidate differential metabolites ranked by VIP score and log2 fold change. Point size indicates VIP score, and color indicates the direction of change. PCs, PEs, monoacylglycerols, and other lipid-related metabolites were prominent among the discriminating features. ***P<0.001.

The candidate differential metabolites were subjected to hierarchical clustering analysis (**Figure 2a**), which revealed a substantial separation tendency in the overall metabolic profiles of the PCOS group and the control group. Phospholipids and lipid-related compounds made up the majority of the top 50 metabolites chosen based on VIP values, which demonstrated consistent change patterns between the two groups. The volcano figure (**Figure 2b**) further supported this pattern, with non-significant metabolites represented as gray dots, significantly downregulated metabolites as blue dots, and markedly increased metabolites as red dots. The majority of the difference metabolites were phospholipids, indicating that lipid remodeling and glycerophospholipid metabolism were major factors in the metabolic alterations of amniotic fluid in PCOS-related pregnancies.

### 3.5 Top–ranked differential metabolites

Phospholipids and lipid-related compounds, such as PC(16:0/18:1(9Z)), PC(P–18:0/18:1(9Z)), MG(16:0/0:0/0:0), and arachidonoyl ethanolamine, are the top metabolites when sorted by fold change and VIP value (**Figure 2c**). This emphasizes the significance of lipid remodeling and glycerophospholipid metabolism in amniotic fluid from pregnant women with PCOS.

### 3.6 Differences in steroid metabolites

**Figure 3:**
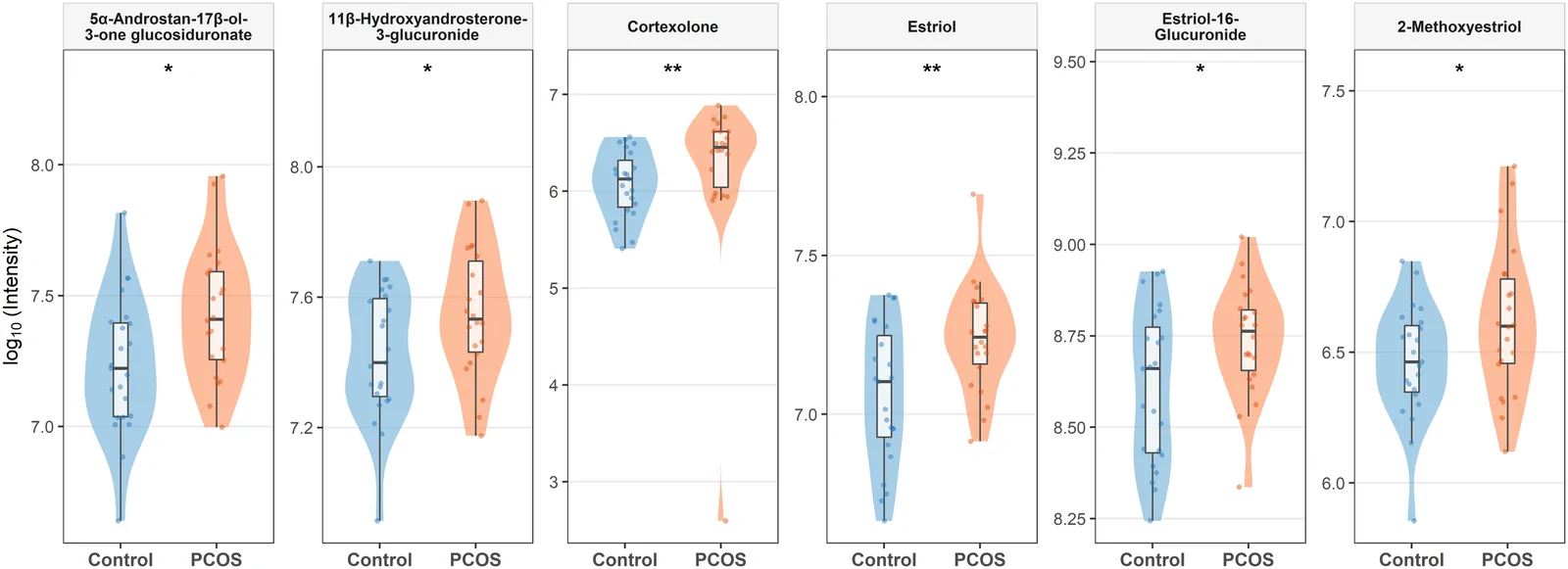
Steroid hormone-related metabolites in amniotic fluid. Violin plots show log2-transformed relative intensities of six steroid hormone-related metabolites in control and polycystic ovary syndrome amniotic fluid. These included three androgen-related metabolites, 5α-androstan-17β-ol-3-one glucosiduronate, 11β-hydroxyandrosterone-3-glucuronide, and cortexolone, and three estrogen-related metabolites, estriol, estriol-16-glucuronide, and 2-methoxyestriol. Boxplots show medians and interquartile ranges, and points represent individual samples. P values for the six metabolites were 0.0131, 0.0353, 0.0084, 0.0039, 0.0224, and 0.0462, respectively. *P < 0.05; **P < 0.01.

From the candidate differential metabolites, we separated the steroid metabolism-related potential differential metabolites into three categories based on their functions: direct androgen metabolites (direct steroids), indirect steroid metabolites, and estrogen metabolites. The overall activity of the steroid metabolic network was evaluated using estrogen metabolites, which are downstream of androgen metabolism. The abundance of these metabolites in the PCOS and control groups is shown as violin plots overlaid with box plots (**Figure 3**), with significant differences between groups marked as *p < 0.05 and **p < 0.01. Three direct androgen metabolites and three estrogen metabolites were significantly upregulated in PCOS amniotic fluid, suggesting that the metabolic features of maternal hyperandrogenism may be reflected in fetal amniotic fluid.

### 3.7 Differential metabolite KEGG pathway enrichment analysis

The KEGG pathway analysis (**Supplementary Figure 5**) demonstrates that the main metabolic alterations in PCOS amniotic fluid are based on lipid metabolism. The sphingolipid signaling pathway and glycerophospholipid metabolism were identified as important nodes with substantial p values (P < 0.05) and strong network impact. These findings suggest that the metabolic anomalies found in PCOS amniotic fluid are focused in lipid-related activities rather than being dispersed randomly throughout pathways. Detailed KEGG enrichment results are provided in **Supplementary Table 4**.

### 3.8 Lipid pathway analysis highlights membrane lipid remodeling and fatty acid metabolism

**Figure 4:**
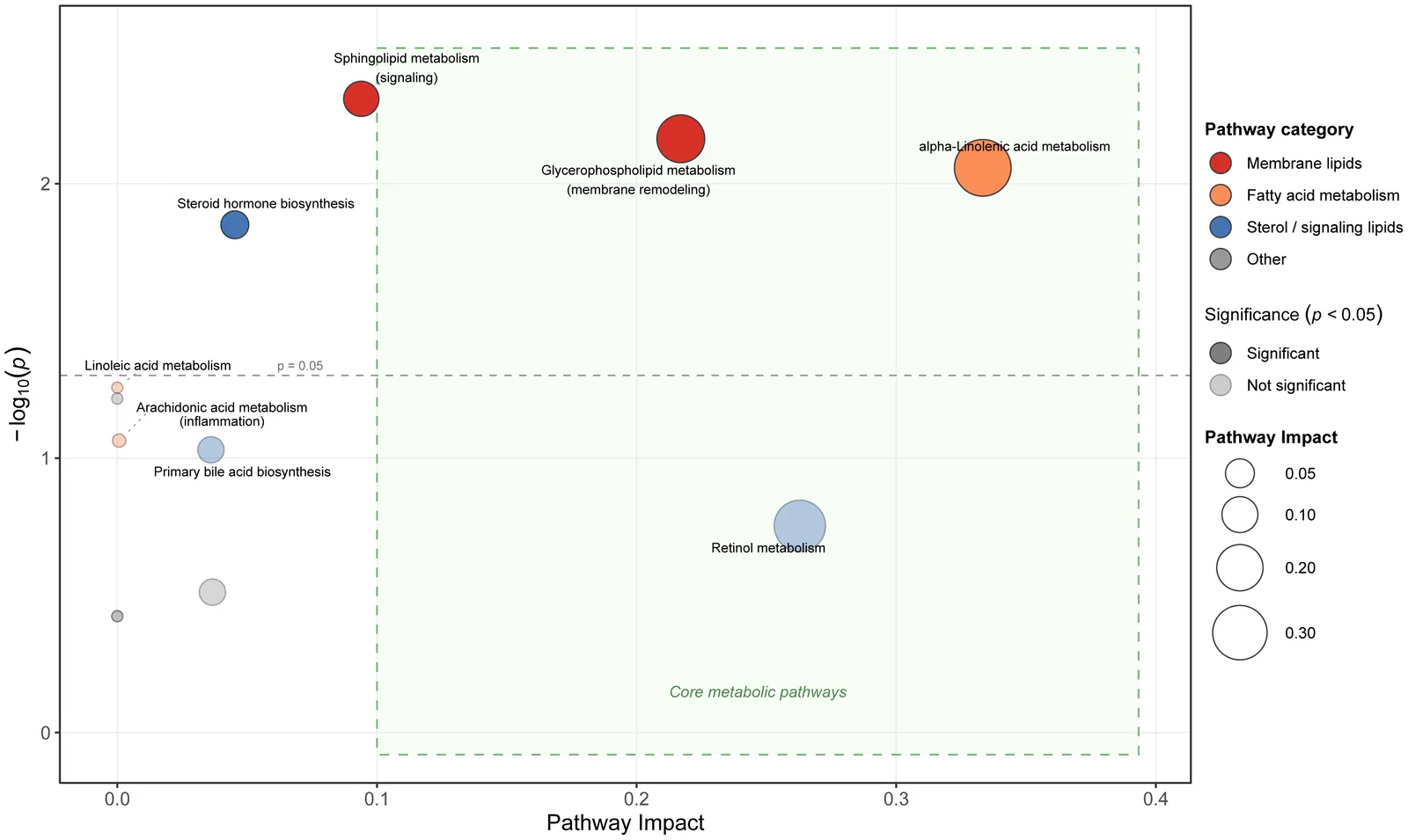
Pathway enrichment and topology analysis of differential lipid metabolites in PCOS amniotic fluid. Pathway analysis was performed using MetaboAnalyst. The x-axis indicates pathway impact, and the y-axis shows statistical significance as −log10(P). Circle size reflects pathway impact, and colors indicate pathway classifications. Glycerophospholipid metabolism, sphingolipid metabolism, and α-linolenic acid metabolism were the major altered pathways. α-Linolenic acid metabolism showed significant enrichment (Raw p = 0.009), steroid hormone biosynthesis showed low pathway impact (Impact = 0.045), retinol metabolism showed high pathway impact despite non-significant enrichment (Raw p = 0.177, Impact = 0.263), and primary bile acid synthesis showed borderline enrichment (Raw p = 0.093).

Considering the central role of lipid metabolites in membrane structure and signal regulation, we performed a targeted pathway analysis with MetaboAnalyst. A total of 12 pathways were identified and grouped into three functional modules: membrane lipid metabolism, fatty acid metabolism, and sterol/signaling lipid metabolism (**Figure 4**). The purpose of this research was to identify the areas of the lipid network that were most strongly implicated, going beyond global pathway enrichment.

Glycerophospholipid metabolism and sphingolipid metabolism were both markedly enriched in the membrane lipid metabolism module. The metabolism of glycerophospholipids had the highest Impact value, and the differential metabolites mostly mapped to important topological nodes, suggesting that cell membrane phospholipids are actively remodeling. The sphingolipid signaling pathway was also significantly disrupted, as seen by the slightly smaller impact but strongest statistical significance of sphingolipid metabolism.

Selective enrichment was observed in the fatty acid metabolism module. Linoleic acid metabolism and arachidonic acid(AA) metabolism were not substantially enriched, although the latter still exhibited a marginal trend consistent with its role in inflammatory regulation. However, alpha–linolenic acid metabolism emerged as a core altered pathway. Enrichment was more diverse in the sterol and signaling lipid module. The biosynthesis of steroid hormones was statistically significant, but the pathway impact was limited. Conversely, retinol metabolism exhibited the opposite pattern, indicating that a small number of differential metabolites may target critical nodal sites. Borderline enrichment was observed in primary bile acid synthesis. In summary, these results suggest that the lipid metabolism network is primarily affected by differential lipid metabolites along two functional axes: membrane lipid remodeling, which is characterized by glycerophospholipid and sphingolipid metabolism, and polyunsaturated fatty acid metabolism, which is centered on alpha–linolenic acid metabolism. Additionally, there are modifications in steroid- and retinol-related signaling.

### 3.9 Identification of core metabolite signatures in PCOS amniotic fluid

In the overall cohort, untargeted metabolomic profiling identified 193 differential metabolites (DEMs) between the PCOS and control groups. We then repeated the analysis after excluding pregnancies with complications, twin gestations, or IVF conception to test whether the results were affected by clinical heterogeneity. These sensitivity analyses identified 274, 232, and 210 DEMs, respectively. A comparison of the four analyses revealed 89 metabolites that were consistently altered, and these were taken forward as the core metabolite set for subsequent analyses. The 89 consistently altered metabolites are listed in **Supplementary Table 5**.

**Figure 5:**
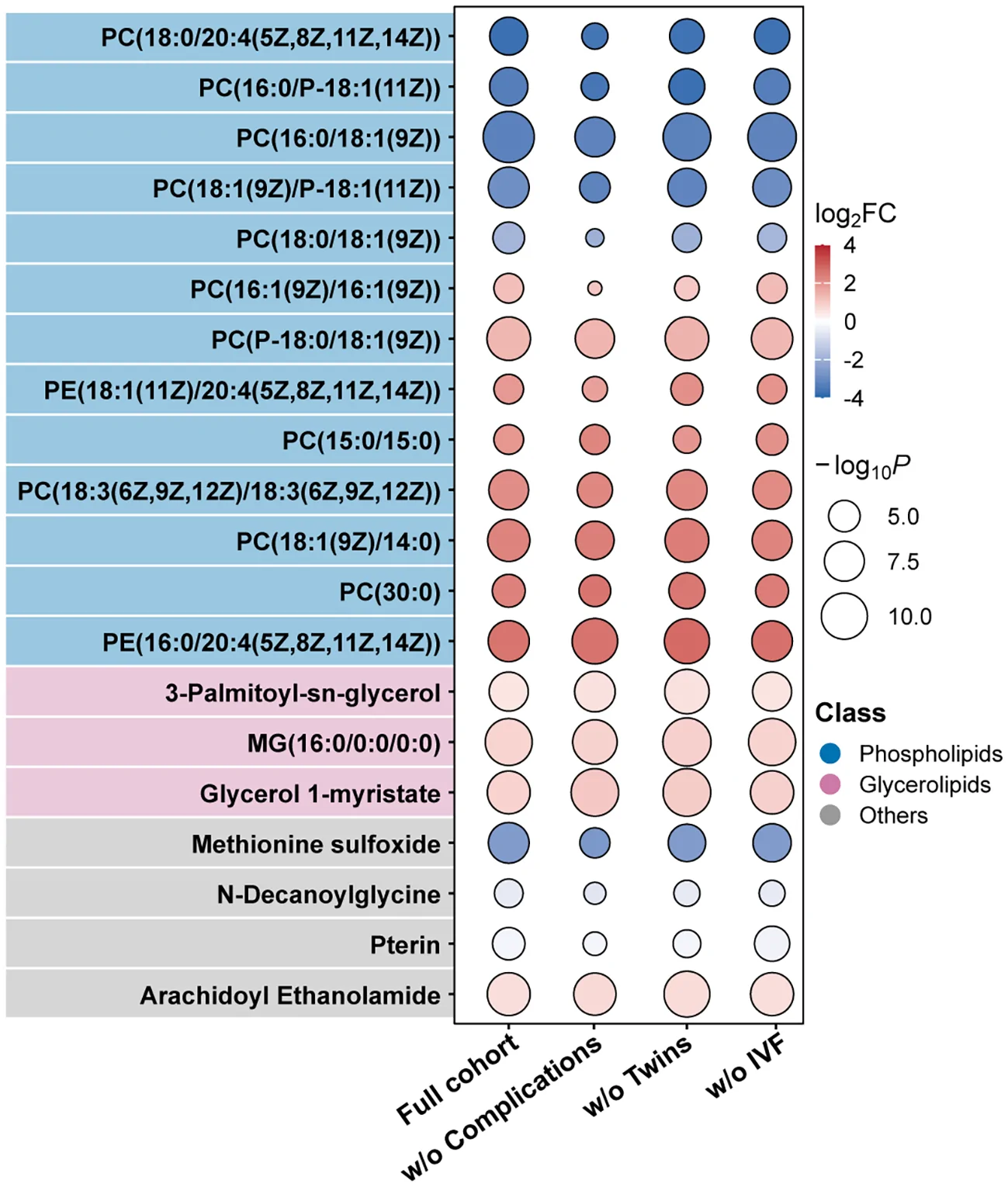
Robustness of the top 20 core differential metabolites across sensitivity analyses. The bubble plot shows the top 20 metabolites retained in the whole-cohort analysis and in sensitivity analyses excluding pregnancies with complications, twin gestations, or IVF conception. Bubble size indicates −log10 P value, and color indicates log2 fold change. Translucent bubbles indicate non-significant changes. The whole-cohort analysis identified 193 differential metabolites. The three sensitivity analyses identified 274, 232, and 210 differential metabolites, respectively, and their overlap defined 89 core metabolites. Phospholipids accounted for 13 of the top 20 core metabolites. Representative changes included decreased PC(16:0/18:1(9Z)) (log2FC = −3.24, P = 4.36 × 10^-13^), decreased PC(18:0/20:4(5Z,8Z,11Z,14Z)) (log2FC = −3.72, P = 1.50 × 10^-7^), and increased PE(16:0/20:4(5Z,8Z,11Z,14Z)) (log2FC = 2.61, P = 1.19 × 10^-8^). All 20 metabolites showed concordant directions across sensitivity analyses. In the complication-excluded analysis, all remained at P < 0.001 except PC(16:1(9Z)/16:1(9Z)) (P < 0.01).

The top 20 core metabolites showed highly concordant expression patterns across all analytical conditions (**Figure 5**). Phospholipids predominated, particularly phosphatidylcholine (PC) and phosphatidylethanolamine (PE) species. Marked reductions in PC(16:0/18:1(9Z)) and PC(18:0/20:4(5Z,8Z,11Z,14Z)), together with elevation of PE(16:0/20:4(5Z,8Z,11Z,14Z)),may indicate disrupted membrane phospholipid homeostasis and support abnormal phospholipid remodeling through the Kennedy pathway and Lands cycle (28–30). In addition, MG(16:0/0:0/0:0) and glycerol 1-myristate were consistently elevated, suggesting enhanced glycerolipid turnover. The stable direction and significance of these metabolites across sensitivity analyses indicate that these alterations are robust metabolic features of PCOS amniotic fluid rather than secondary effects of clinical heterogeneity.

### 3.10 PCOS-associated metabolite changes remained robust across sensitivity analyses

Pairwise correlation analysis of log2FC values for the 89 core metabolites between the full cohort and each sensitivity analysis showed extremely high consistency (**Supplementary Figure 6**). The strongest concordance was observed in the IVF-excluded analysis, followed by the twin-excluded analysis, while the complication-excluded analysis also retained a high correlation despite substantial reduction in sample size. In all comparisons, metabolites clustered closely around the y = x diagonal, and no metabolite showed reversal of direction. These findings indicate that pregnancy complications, twin gestation, and IVF caused only minor quantitative fluctuations without altering the overall metabolic pattern, supporting the robustness of the identified PCOS-associated metabolic features.

In accordance with this, the UpSet diagram (**Supplementary Figure 7**) illustrated the overlap of DEMs among the four analyses. The largest intersection set was formed by 135 metabolites (38.9%) that were significant in all four analyses, out of the 347 metabolites that were significant in at least one analysis. This suggests that nearly 40% of DEMs were not influenced by the sample selection strategy, further supporting the stability of the core metabolites across analytical conditions. The complication-excluded analysis showed the largest number of unique DEMs, with 68 metabolites (19.6%) unique to this subset, consistent with its highest total DEM yield. Furthermore, 34 metabolites were specifically shared between the complication-excluded and twin-excluded analyses, indicating that these two clinical factors may influence metabolite detection sensitivity through partially overlapping mechanisms. In contrast, the full-cohort analysis contained only 8 unique DEMs, suggesting that its coverage was already relatively adequate. The value of multi-level screening, which includes VIP and FDR thresholds, in enhancing biomarker reliability is further illustrated by the disparity between the 135 metabolites shared across analyses and the 89 final core metabolites.

### 3.11 Lipid-related metabolites consistently dominated across analyses

To determine whether specific metabolite classes were selectively represented in the core metabolic signature, we examined both overall class composition and class enrichment across the four analyses (**Supplementary Figure 8**). Class composition remained largely consistent, with fatty acid derivatives and phospholipids as the major categories in all analyses. In the full cohort, fatty acid derivatives were the most abundant class, followed by phospholipids, whereas lysophospholipids, sphingolipids, glycerolipids, and triglycerides were less abundant but consistently present. The total number of DEMs increased significantly after the exclusion of pregnancies with complications, primarily due to the expansion of lysophospholipids and fatty acid derivatives, while the number of phospholipids remained unchanged. This suggests that the exclusion of patients with complications may have increased metabolic dispersion within the PCOS group and masked some differential signals. In contrast, the twin-excluded and IVF-excluded analyses showed class distributions similar to the full cohort, further supporting the limited impact of these two factors on metabolic profiles. Notably, lipid-related classes consistently accounted for 40–50% of DEMs across all four analyses, consistent with previous metabolomic studies in PCOS (31, 32).

### 3.12 Representative core metabolites revealed phospholipid remodeling in PCOS amniotic fluid

**Figure 6:**
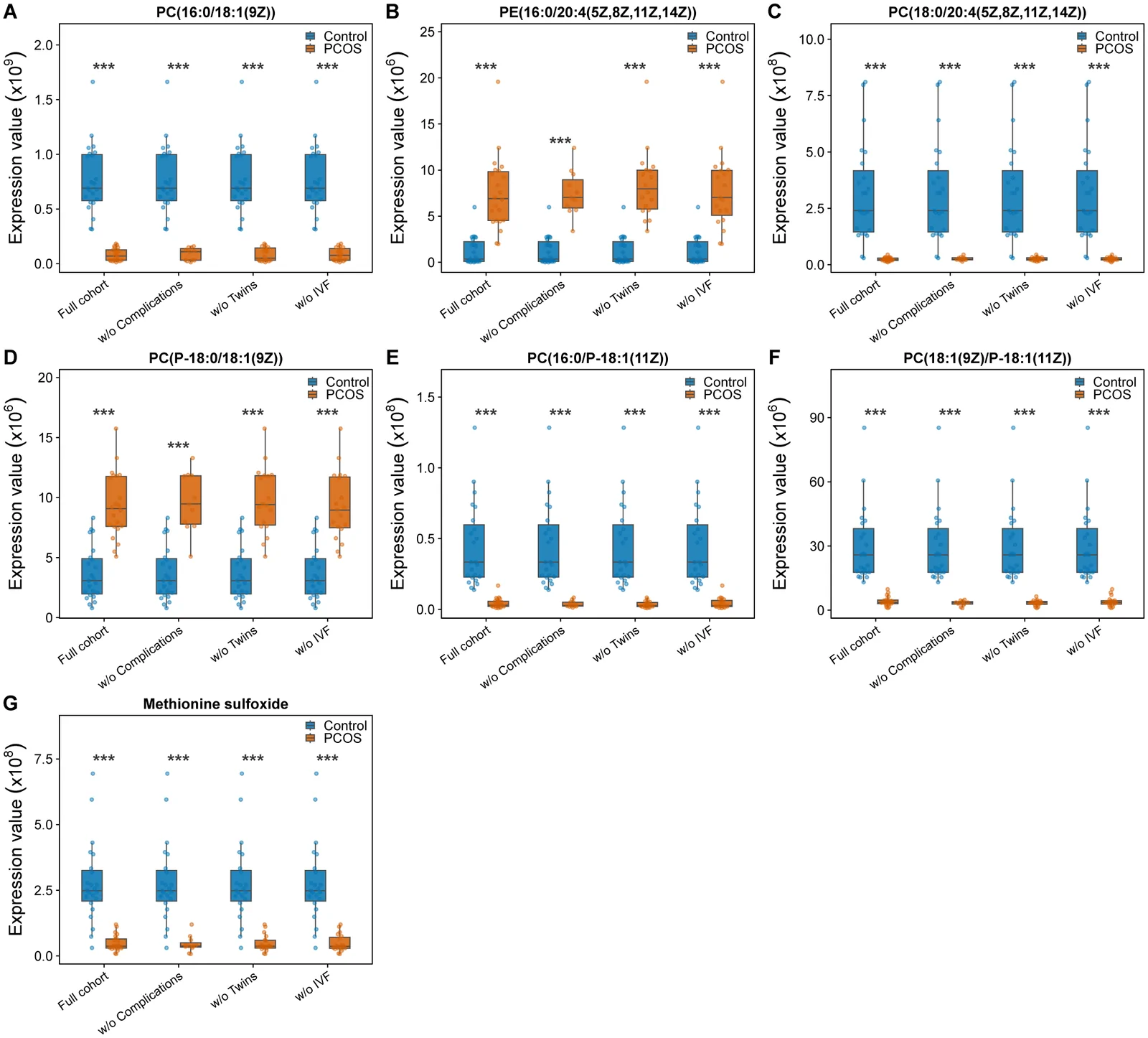
Seven representative core metabolites across sensitivity analyses. **(A)** PC(16:0/18:1(9Z)); **(B)** PE(16:0/20:4(5Z,8Z,11Z,14Z)); **(C)** PC(18:0/20:4(5Z,8Z,11Z,14Z)); **(D)** PC(P–18:0/18:1(9Z)); **(E)** PC(16:0/P–18:1(11Z)); **(F)** PC(18:1(9Z)/P–18:1(11Z)); and **(G)** methionine sulfoxide. Box plots compare six phospholipid species and methionine sulfoxide between the control and polycystic ovary syndrome groups in the whole cohort and in sensitivity analyses. Points represent individual samples. Boxes indicate interquartile ranges, and whiskers extend to 1.5 × IQR. All seven metabolites remained significantly different between groups in all analyses (all P < 0.001). In the whole cohort, PC(16:0/18:1(9Z)) decreased from 7.72 × 10^8^ in controls to 8.19 × 10^7^ in polycystic ovary syndrome samples (P = 4.36 × 10^-13^). PC(18:0/20:4(5Z,8Z,11Z,14Z)), PC(16:0/P–18:1(11Z)), and PC(18:1(9Z)/P–18:1(11Z)) were also reduced (P = 1.50 × 10^-7^, 1.14 × 10^-7^, and 1.71 × 10^-8^, respectively). PE(16:0/20:4(5Z,8Z,11Z,14Z)) increased from 1.23 × 10^6^ to 7.47 × 10^6^ (P = 1.19 × 10^-8^). PC(P–18:0/18:1(9Z)) was increased (log2FC = 1.35), while methionine sulfoxide decreased from 2.77 × 10^8^ to 4.85 × 10^7^ (P = 1.71 × 10^-8^). Control, n = 23; polycystic ovary syndrome: whole cohort, n = 22; excluding complications, n = 11; excluding twins, n = 18; excluding IVF, n = 19. ***P < 0.001.

Subsequently, we investigated individual core metabolites that were representative. The control and PCOS groups exhibit individual–level expression differences for seven representative core metabolites, including six phospholipid species and methionine sulfoxide, as illustrated in **Figure 6**. In the full cohort and all sensitivity analyses, all seven metabolites achieved a P-value of less than 0.001.

PC(16:0/18:1(9Z)) was approximately one–tenth as abundant in the PCOS group as in controls. This PC species is a major component of mammalian cell membranes, and its marked reduction is associated with decreased *de novo* PC synthesis or impaired hepatic transport (29, 33). PC(18:0/20:4(5Z,8Z,11Z,14Z)), which contains an AA side chain, was also downregulated. Its reduction may accelerate AA release via the phospholipase A_2_ pathway, thereby promoting prostaglandin and leukotriene generation (30). Both PC(16:0/P–18:1(11Z)) and PC(18:1(9Z)/P–18:1(11Z)) are plasmalogens that exhibited downregulation in the PCOS cohort. Plasmalogens are reactive oxygen species scavengers, and their reduction is consistent with an increased oxidative stress burden (34), which supports the notion that antioxidant defenses are compromised in PCOS.

PE(16:0/20:4(5Z,8Z,11Z,14Z)) was approximately six–fold higher in the PCOS group. An elevated PE/PC ratio is associated with altered membrane fluidity and activation of apoptotic signaling pathways (28, 35). Upregulation of PC(P–18:0/18:1(9Z)), an ether phospholipid, suggests compensatory activation of certain ether phospholipid synthesis pathways. Methionine sulfoxide was reduced to approximately one–fifth of control levels in the PCOS group. As a product of methionine oxidation by reactive oxygen species, this change may reflect altered methionine sulfoxide reductase activity or an imbalance in the methionine cycle itself. The separation between groups for these seven metabolites did not change appreciably after excluding complications, twins, or IVF, further confirming the PCOS specificity of these differences.

### 3.13 Phospholipids were selectively enriched among core metabolites

**Figure 7:**
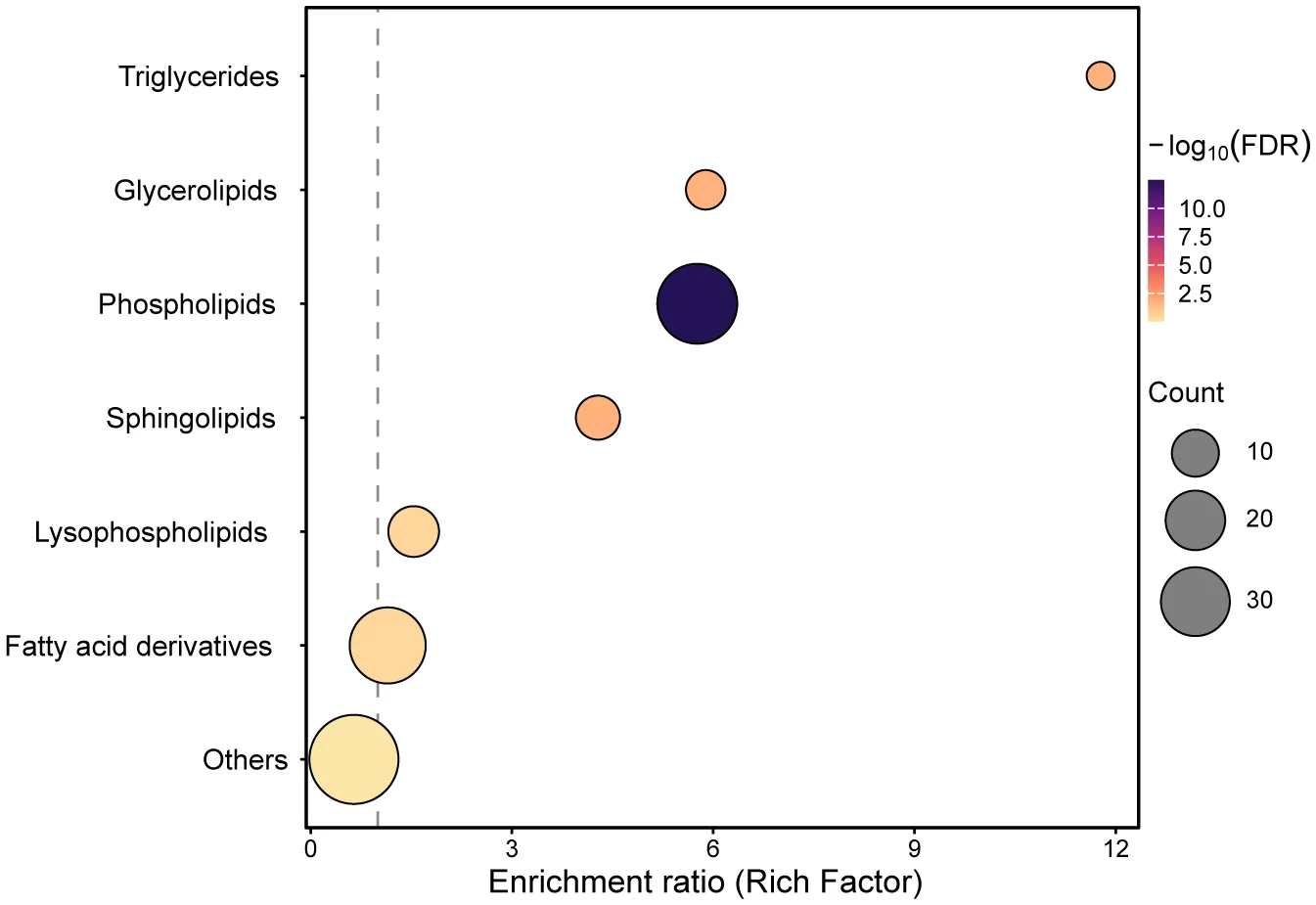
Class enrichment analysis of the 89 core metabolites relative to the full metabolome (1,048 metabolites). Fisher’s exact test was used to assess enrichment of the 89 core metabolites across chemical classes against the full metabolome background. Dot size indicates the number of core metabolites in each class, and color depth represents −log10(FDR). The vertical dashed line marks an enrichment ratio of 1. Phospholipids showed the strongest enrichment (Rich Factor = 5.76, FDR = 3.17 × 10^-13^), with 23 of 47 background phospholipids retained in the core set. Triglycerides had the highest enrichment ratio (Rich Factor = 11.78, FDR = 0.018), although only two species were detected, both downregulated in PCOS (log2FC = −5.73 and −3.62). Glycerolipids (Rich Factor = 5.89, FDR = 0.018) and sphingolipids (Rich Factor = 4.28, FDR = 0.018) were also significantly enriched; SM(d18:2(4E,14Z)/16:0) was upregulated (log2FC = 3.50). In contrast, fatty acid derivatives (20 core metabolites; Rich Factor = 1.15, FDR = 0.320) and lysophospholipids (Rich Factor = 1.54, FDR = 0.264) were not significantly enriched.

Using the full metabolome (1,048 metabolites) as background, Fisher’s exact test was used to assess class enrichment of the 89 core metabolites (**Figure 7**). Phospholipids showed the most robust enrichment, with 23 of 47 background phospholipids (48.9%) retained in the core set, indicating that phospholipid remodeling is a central feature of PCOS-associated metabolic disturbance. Triglycerides exhibited the highest enrichment ratio, despite the limited number of detectable species. Furthermore, both discovered TGs were significantly downregulated, indicating selective remodeling of specific TG subspecies rather than a reduction in total triglyceride levels. Glycerolipids and sphingolipids were also significantly enriched, supporting broader disruption of lipid remodeling pathways. By contrast, fatty acid derivatives ranked second in number in the core set (20 metabolites), but their enrichment ratio was only 1.15, indicating no significant enrichment. This probably reflects their high background proportion in the full metabolome (205/1,048, 19.6%), which was comparable to their proportion in the core set (22.5%). This suggests that their background composition has not been selectively enhanced. Lysophospholipids likewise showed no significant enrichment. Overall, phospholipid remodeling appears as the primary metabolic alteration in PCOS. Changes in triglycerides and sphingolipids are secondary features, while widespread fatty acid metabolism mainly reflects the general response of the lipid system (31, 32, 36).

### 3.14 Clinical associations of representative metabolites and PC/PE imbalance

**Figure 8:**
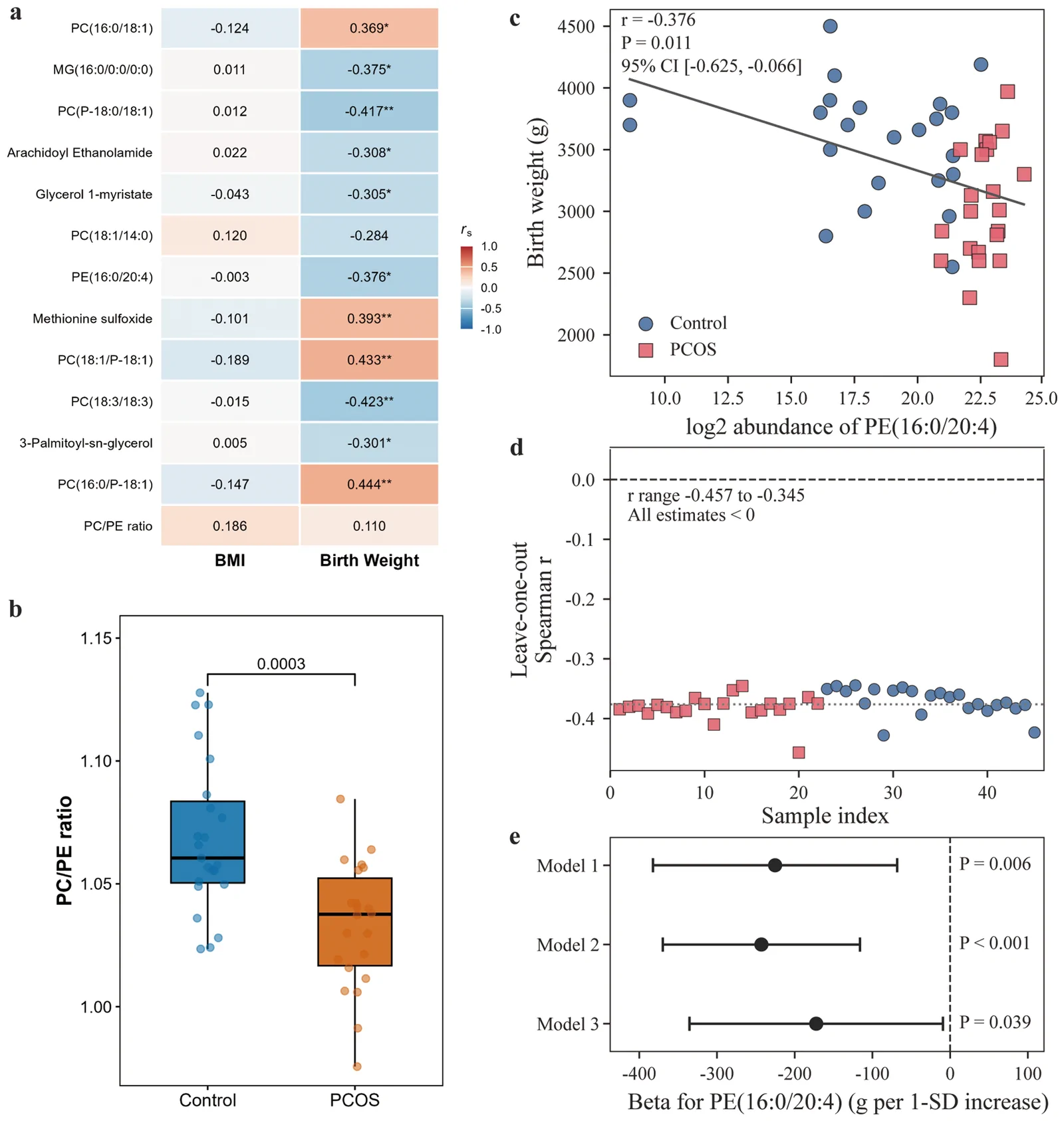
Associations of selected amniotic fluid metabolites with maternal BMI at delivery and neonatal birth weight, group differences in the PC/PE ratio, and sensitivity analyses of PE(16:0/20:4). **(a)** Spearman correlation coefficients are shown for 12 core metabolites and the phosphatidylcholine/phosphatidylethanolamine ratio with maternal BMI at delivery and neonatal birth weight. None of the metabolites or the PC/PE ratio showed significant correlations with BMI at delivery. Birth weight was positively correlated with PC(16:0/P–18:1) (r_s_= 0.444), PC(18:1/P–18:1) (r_s_= 0.433), methionine sulfoxide (r_s_= 0.393), and PC(16:0/18:1) (r_s_= 0.369). Birth weight was negatively correlated with PC(18:3/18:3) (r_s_= −0.423), PC(P–18:0/18:1) (r_s_= −0.417), PE(16:0/20:4), MG(16:0/0:0/0:0), arachidonoyl ethanolamide, glycerol 1-myristate, and 3-palmitoyl-sn-glycerol (r_s_= −0.301 to −0.376). **(b)** The phosphatidylcholine/phosphatidylethanolamine ratio was lower in the polycystic ovary syndrome group than in controls (median 0.62 vs. 1.15; Mann–Whitney U test, P = 0.0003; n_control = 23, n_polycystic ovary syndrome = 22). Points represent individual samples, and boxes indicate medians and interquartile ranges. *P < 0.05; **P < 0.01. **(c)** Scatterplot showing the relationship between the log_2_-transformed abundance of PE(16:0/20:4) and birth weight in the full cohort of 45 pregnancies. PE(16:0/20:4) was negatively correlated with birth weight (Spearman’s r = −0.376, P = 0.0109). The bootstrap 95% confidence interval based on 10,000 resamples was −0.625 to −0.066. Blue circles represent control pregnancies, red squares represent PCOS pregnancies, and the solid line indicates the fitted trend. **(d)** Leave-one-out sensitivity analysis. Spearman’s correlation coefficients were recalculated after sequentially excluding each of the 45 samples. All estimates remained negative, ranging from −0.457 to −0.345. Each point represents the estimate obtained after excluding one sample, with its color indicating the group of the omitted sample. The dashed horizontal line denotes r = 0. **(e)** Linear regression estimates for the difference in birth weight associated with each 1-SD increase in PE(16:0/20:4) abundance. Model 1 was unadjusted (β = −225.3 g, 95% CI: −382.4 to −68.1, P = 0.006). Model 2 was adjusted for gestational age at delivery, fetal sex, and plurality (β= −242.9 g, 95% CI: −369.7 to −116.1, P < 0.001). Model 3 was additionally adjusted for PCOS status (β= −172.4 g, 95% CI: −335.4 to −9.3, P = 0.039). Points indicate regression coefficients, horizontal lines indicate 95% confidence intervals, and the dashed vertical line denotes β= 0.

To investigate the relationships between the major metabolic alterations and maternal anthropometric or neonatal growth parameters, we considered pre-pregnancy BMI, BMI at delivery, gestational weight change, and neonatal birth weight. Pre-pregnancy BMI and BMI at delivery were comparable between the PCOS and control groups (**Supplementary Table 1**). Gestational weight change was not included as an independent covariate because it represents a composite pregnancy-related phenotype influenced by baseline maternal characteristics and gestational factors. After identification of the core metabolic signature, we further examined the associations of these metabolic features with maternal adiposity and neonatal growth outcomes (**Figure 8a**).

Instead of selecting metabolites solely according to rank order, we selected 12 representative metabolites from the final core set to cover the major altered metabolic features, including PC/PE remodeling, AA-containing phospholipid remodeling, glycerolipid turnover, fatty-acid-derived lipid signaling, and oxidative metabolism. To evaluate whether maternal adiposity contributed to these metabolic alterations, pre-pregnancy BMI was first examined. Pre-pregnancy BMI did not differ significantly between the groups (**Supplementary Figure 10**). None of the 12 representative metabolites or the PC/PE ratio showed significant correlations with pre-pregnancy BMI. Furthermore, ordinary least-squares regression models were fitted with each metabolic feature as the dependent variable and PCOS status as the primary predictor, with and without adjustment for pre-pregnancy BMI. After adjustment, the PCOS coefficient retained the same direction for all metabolic features and remained statistically significant across all outcomes, indicating that the identified metabolic alterations were unlikely to be explained by differences in maternal adiposity.

We next examined the relationships between representative metabolic features and BMI at delivery, which more closely reflected the maternal metabolic state at the time of late-gestation amniotic-fluid collection. None of the 12 representative metabolites or the PC/PE ratio showed significant correlation with BMI at delivery. In contrast, multiple metabolites showed significant associations with birth weight, including both positive and negative correlations, suggesting a closer link between lipid metabolic alterations and fetal growth outcomes. Positive correlations included PC(16:0/P–18:1), which showed the strongest association, followed by PC(18:1/P–18:1), methionine sulfoxide, and PC(16:0/18:1). Negative correlations included PC(18:3/18:3), PC(P–18:0/18:1), PE(16:0/20:4), MG(16:0/0:0/0:0), arachidonoyl ethanolamide, glycerol 1–myristate, and 3–palmitoyl–sn–glycerol. The PC/PE ratio exhibited no correlation with BMI at delivery or birth weight despite clear group differences, suggesting that the observed phospholipid imbalance was not strongly related to maternal BMI or neonatal birth weight in the present cohort. Associations between selected metabolites and neonatal birth weight are shown in **Supplementary Table 6**.

Among these metabolites, PE(16:0/20:4) showed a significant inverse association with birth weight, prompting further assessment of its robustness. This relationship remained consistent across bootstrap resampling (**Figure 8c**), leave-one-out analysis (**Figure 8d**), multivariable regression (**Figure 8e**), and analyses restricted to singleton, non-IVF, and complication-free pregnancies (**Supplementary Table 11**). It also remained significant after adjustment for gestational age at delivery, fetal sex, plurality, and PCOS status, lending further support to the observed association in the present cohort (**Figure 8e**).

Because opposite changes in PC and PE emerged repeatedly across analyses, we further examined the PC/PE ratio as a summary index of membrane phospholipid balance (**Figure 8b**). The PC/PE ratio was significantly lower in the PCOS group than in controls. This finding suggests a change in the relative amounts of phosphatidylcholine and phosphatidylethanolamine in the amniotic fluid of pregnancies with PCOS. Both PC and PE are core components of cell membranes. An altered PC/PE ratio may therefore reflect membrane phospholipid remodeling or an abnormality in the rate of phospholipid conversion through the Kennedy pathway, although the exact mechanisms require further validation.

### 3.15 Selected amniotic fluid lipids showed robust associations with birth weight

**Figure 9:**
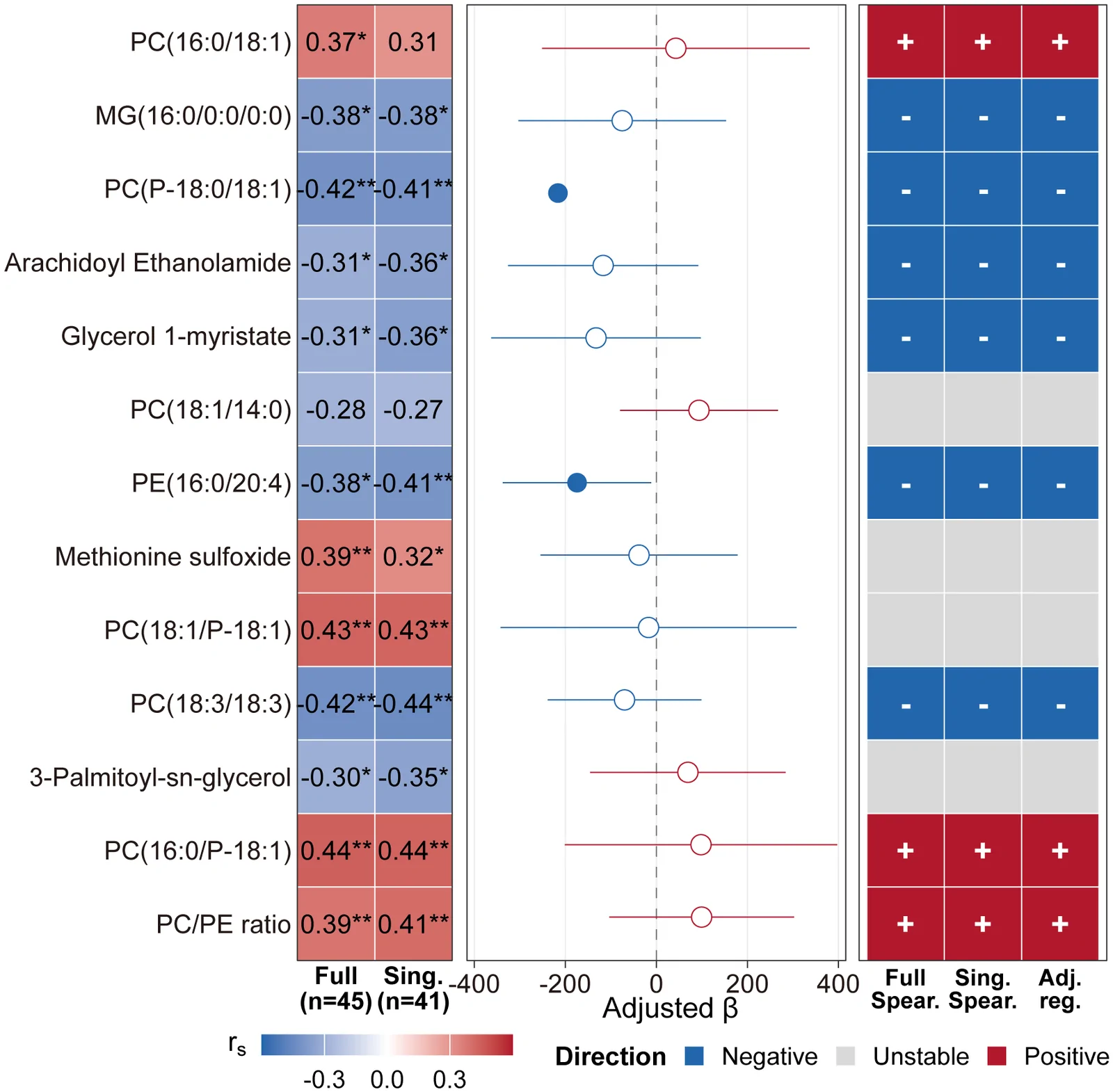
Associations between selected amniotic fluid metabolites and neonatal birth weight. Spearman correlation analyses were performed in the whole cohort and in the singleton-only cohort after excluding four twin pregnancies. The left panel shows correlation coefficients. In the whole cohort, positive correlations with birth weight were observed for PC(16:0/18:1) (r = 0.37), PC(18:1/14:0) (r = 0.28), methionine sulfoxide (r = 0.39), PC(18:1/P–18:1) (r = 0.43), PC(16:0/P–18:1) (r = 0.44), and the phosphatidylcholine/phosphatidylethanolamine ratio (r = 0.39). Negative correlations were observed for MG(16:0/0:0/0:0) (r = −0.38), PC(P–18:0/18:1) (r = −0.42), arachidonoyl ethanolamide (r = −0.31), glycerol 1-myristate (r = −0.31), PE(16:0/20:4) (r = −0.38), PC(18:3/18:3) (r = −0.42), and 3-palmitoyl-sn-glycerol (r = −0.30). The middle panel shows β coefficients and 95% confidence intervals from linear regression models adjusted for polycystic ovary syndrome status, gestational age at delivery, fetal sex, and fetal number. After adjustment, PC(P–18:0/18:1) remained associated with lower birth weight (β = −216.32 g, 95% CI: −404.96 to −27.68, P = 0.026), as did PE(16:0/20:4) (β = −174.37 g, 95% CI: −337.48 to −11.25, P = 0.037). The right panel summarizes the direction of associations across whole-cohort Spearman analysis, singleton-only Spearman analysis, and adjusted regression analysis. Asterisks indicate statistical significance.

We further examined the associations between selected amniotic fluid metabolites and neonatal birth weight (**Figure 9**). In Spearman correlation analyses, most metabolites showed comparable correlation directions in the full cohort and in the singleton-only cohort, indicating that the observed associations were not mainly driven by twin pregnancies. The PC/PE ratio and several PC species were positively correlated with birth weight, whereas PC(P-18:0/18:1), PE(16:0/20:4), PC(18:3/18:3), MG(16:0/0:0/0:0), arachidoyl ethanolamide, and glycerol 1-myristate showed negative correlations.

After adjustment for PCOS status, gestational age at delivery, fetal sex, and fetal number, most associations were attenuated. Notably, PC(P-18:0/18:1) and PE(16:0/20:4) remained significantly and negatively associated with birth weight. These findings suggest that a subset of amniotic fluid lipid metabolites shows relatively stable associations with neonatal birth weight, with PC(P-18:0/18:1) and PE(16:0/20:4) showing the most robust negative associations after covariate adjustment.

### 3.16 Selective placental transcriptomic support for amniotic fluid lipid remodeling

**Figure 10:**
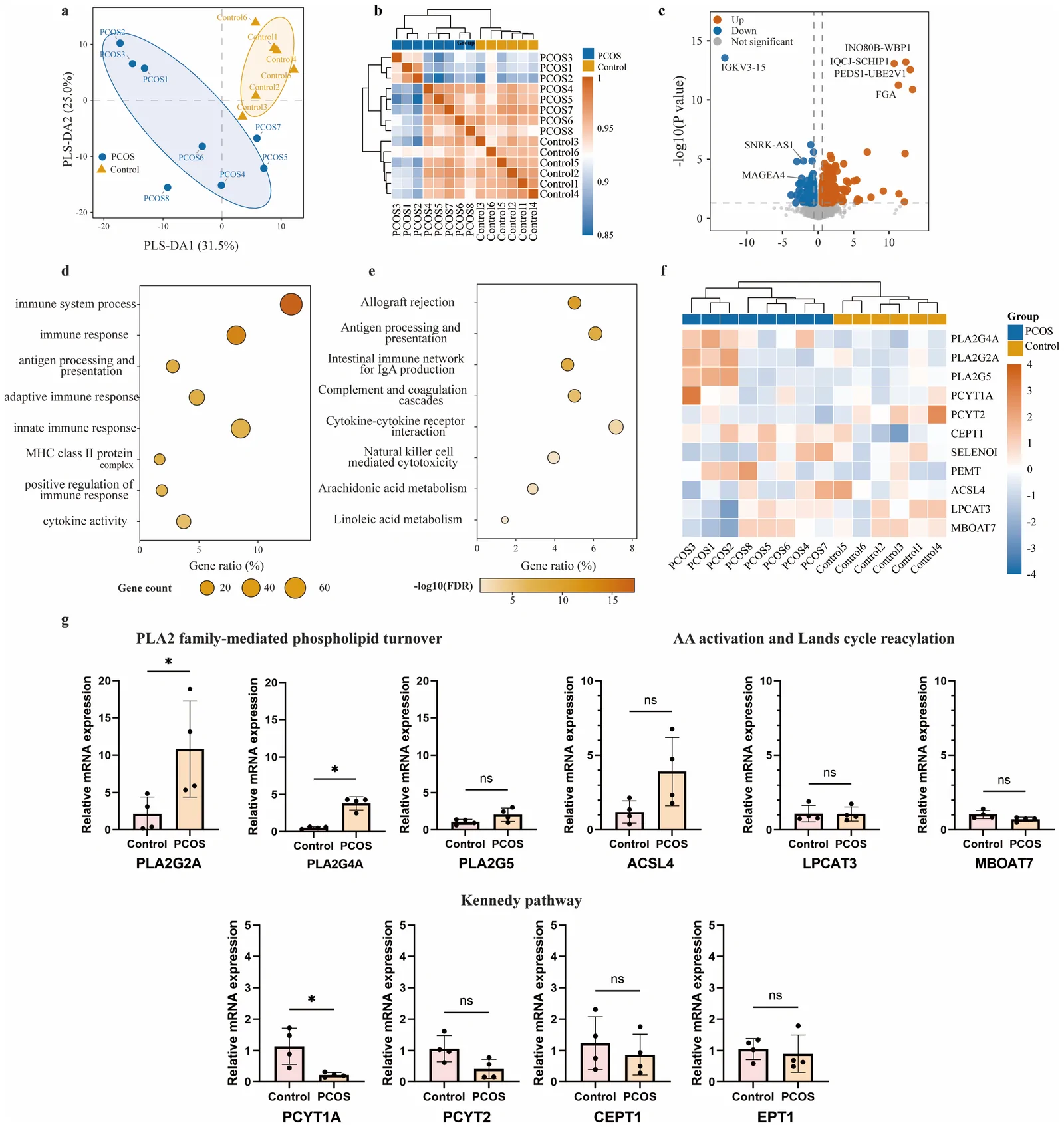
Placental transcriptomic analysis and targeted RT-qPCR assessment of phospholipid metabolism-related genes in PCOS pregnancies. **(a)** Partial least-squares discriminant analysis (PLS-DA) of placental transcriptomic profiles from women with PCOS (n = 8) and controls (n = 6). **(b)** Sample-to-sample correlation matrix with hierarchical clustering. Colors indicate Pearson correlation coefficients. **(c)** Volcano plot of differentially expressed genes between the PCOS and control groups. Genes with P<0.05 and fold change (FC)≥1.5 or ≤0.667 were considered differentially expressed; orange and blue dots indicate upregulated and downregulated genes, respectively, and gray dots indicate genes that did not meet these criteria. **(d, e)** GO and KEGG enrichment analyses of the differentially expressed genes. Bubble size represents gene count or gene ratio, as indicated, and color represents −log10(FDR). **(f)** Heatmap of selected genes involved in PLA2-mediated phospholipid turnover, the Kennedy pathway, and AA activation/Lands cycle reacylation. Expression values were standardized by gene and are shown as Z scores. **(g)** RT-qPCR analysis of selected phospholipid metabolism-related genes in an independent placental cohort, including genes involved in PLA2 family-mediated phospholipid turnover (PLA2G2A, PLA2G4A, and PLA2G5), AA activation and Lands cycle reacylation (ACSL4, LPCAT3, and MBOAT7), and the Kennedy pathway (PCYT1A, PCYT2, CEPT1, and EPT1). The RT-qPCR cohort included four women with PCOS and four controls and did not overlap with the RNA-seq cohort. Relative expression was normalized to ACTB and TBP. Data are presented as mean ± SD. *P < 0.05; ns, not significant.

Because of the limited sample size, the placental analyses were interpreted as exploratory tissue-level contextualization of the amniotic-fluid findings. Since the placenta is the primary interface controlling fetal nutrient and lipid exposure, whereas amniotic fluid reflects integrated metabolic contributions from maternal, fetal, and placental sources, placental transcriptomic analysis was performed to provide tissue-level context for the amniotic-fluid lipidomic findings (37, 38) (**Figure 10a**). RNA sequencing was performed on placental samples from 8 women with PCOS and 6 controls. Quality-control results are shown in **Supplementary Figure 9**, and expression data for selected phospholipid metabolism-related genes are provided in **Supplementary Table 12**. Based on the metabolomic evidence of altered phospholipid metabolism, key genes involved in phospholipid turnover, fatty-acid reacylation, and *de novo* phospholipid synthesis were further examined by RT-qPCR in an independent placental cohort. This targeted analysis was intended to assess selected nodes within the implicated phospholipid metabolic pathways rather than to directly validate RNA-seq-defined DEGs.

PLS-DA showed separation between the PCOS and control groups, while hierarchical clustering of the sample correlation matrix also suggested differences in the overall placental expression profiles (**Figures 10a, b**). Differential expression analysis identified 863 genes, including 605 upregulated and 258 downregulated genes in PCOS placentas (**Figure 10c**). The complete list of differentially expressed genes, together with their fold changes, P values, and adjusted P values, is provided in **Supplementary Table 13**.

GO and KEGG enrichment analyses indicated that immune-related processes represented a prominent component of the placental transcriptional differences. Upregulated genes were enriched in immune response, antigen processing and presentation, MHC class II-related processes, complement and coagulation cascades, cytokine–cytokine receptor interaction, and natural killer cell-mediated cytotoxicity (**Figures 10d, e**). In addition to these immune-related pathways, arachidonic acid metabolism and linoleic acid metabolism were also significantly enriched among the upregulated genes, suggesting that changes in polyunsaturated fatty-acid-related pathways may accompany the broader placental transcriptional alterations.

Given the amniotic-fluid lipidomic findings, including changes in PC/PE balance and AA-containing phospholipid species, we next examined genes involved in PLA2-mediated phospholipid hydrolysis, Kennedy pathway-related PC/PE synthesis, and Lands cycle-related fatty-acyl reacylation. Among these genes, the clearest changes in the RNA-seq dataset were observed in the PLA2 family, with PLA2G4A, PLA2G2A, and PLA2G5 showing significant upregulation in PCOS placentas. As these enzymes are involved in the release of arachidonic and linoleic acids from membrane phospholipids, their expression changes were consistent with the enrichment of arachidonic acid and linoleic acid metabolism. In contrast, changes in genes involved in *de novo* phospholipid synthesis and reacylation were less consistent. PCYT2 showed a nominal decrease but did not meet the fold-change threshold, PEMT and CEPT1 showed only weak trends, PCYT1A showed no clear difference, and ACSL4, LPCAT3, and MBOAT7 were not significantly altered (**Figure 10f**; **Supplementary Table 12**).

These genes were further examined by RT-qPCR in an independent, non-overlapping placental cohort. PLA2G4A and PLA2G2A were significantly upregulated in PCOS placentas, while PLA2G5 showed a nonsignificant upward trend (**Figure 10g**). Among genes involved in the Kennedy pathway, PCYT1A was significantly downregulated, PCYT2 showed a nonsignificant downward trend, and CEPT1 and EPT1 remained unchanged. No significant differences were detected in the Lands cycle-related genes ACSL4, LPCAT3, or MBOAT7 (**Figure 10g**).

Overall, the placental transcriptomic data suggest that immune-related transcriptional changes are a major feature of PCOS placentas, with additional but more selective alterations involving PLA2-associated phospholipid hydrolysis and polyunsaturated fatty-acid metabolism. The concordant changes in PLA2G4A and PLA2G2A across RNA-seq and RT-qPCR provide preliminary supportive tissue-level evidence that is consistent with the amniotic-fluid lipidomic findings. By contrast, changes in PC/PE balance and fatty-acyl remodeling were more evident at the metabolite level than at the placental transcript level.

## 4 Discussion

Amniotic fluid represents a biochemical interface between the mother, placenta, and fetus, and its metabolomic composition provides important information about the intrauterine environment experienced by the fetus before birth. Previous studies have shown that the amniotic fluid metabolome can reflect fetal physiological and pathological states and may capture early metabolic features associated with subsequent disease risk. In the present study, the amniotic fluid metabolic profile differed markedly between women with PCOS and controls, mainly involving membrane phospholipid remodeling, polyunsaturated fatty acid metabolism, and sphingolipid metabolism, together with changes in a small number of steroid-related metabolites. These findings suggest that PCOS-related metabolic disturbances extend beyond the maternal circulation and placenta to the amniotic fluid environment surrounding the fetus.

Phospholipid remodeling was one of the most prominent metabolic features in PCOS-related pregnancies. Several representative PC species were decreased, including PC(16:0/18:1) and the AA-containing species PC(18:0/20:4), whereas the AA-containing PE species PE(16:0/20:4) was increased. The PC/PE ratio was also significantly lower in the PCOS group. PC and PE are major structural phospholipids whose relative abundance is regulated by *de novo* synthesis, PE-to-PC conversion, phospholipid hydrolysis, and fatty acyl-chain remodeling. Therefore, the decrease in PC, increase in PE, and lower PC/PE ratio, particularly the opposite changes in AA-containing PC and PE species, suggest altered PC/PE homeostasis and redistribution of AA-containing phospholipids in PCOS amniotic fluid.

These changes may have implications for membrane biology. PC contributes to the stability of the lipid bilayer, whereas PE regulates membrane curvature, fluidity, and remodeling. Changes in their relative abundance can therefore affect phospholipid organization and organelle membranes properties. Marked PC/PE imbalance has previously been linked to disturbed endoplasmic reticulum calcium homeostasis, cellular stress responses, and mitochondrial dysfunction (39, 40). During pregnancy, placental endoplasmic reticulum and oxidative stress contribute to the pathological processes underlying fetal growth restriction and early-onset preeclampsia (41), while inflammation and oxidative stress in the fetal membranes have been associated with membrane weakening, preterm prelabor rupture of membranes, and preterm birth (42). Because late-pregnancy amniotic fluid is jointly influenced by fetal urine, fetal lung secretions, fetal membrane exchange and substances released from the fetal side of the placenta (43, 44), the lower PC/PE ratio observed in this study may reflect changes in the local membrane lipid environment within the intrauterine compartment. Although the present data cannot identify the specific tissue source or demonstrate impaired membrane function, they suggest disturbed membrane lipid homeostasis within the fetal developmental environment of PCOS pregnancies.

This metabolic pattern may involve selective mobilization of the AA-rich PC pool, impaired maintenance of the PC pool, and redistribution of AA among different phospholipid pools. AA is generally esterified at the sn-2 position of membrane phospholipids and AA-containing PC can undergo PLA2-mediated hydrolysis to release AA for lipid-mediator production. Therefore, the decrease in PC(18:0/20:4) may be related to selective turnover or remodeling of the AA-rich PC pool. Consistent with this interpretation, placental transcriptomic analysis showed significant enrichment of arachidonic acid metabolism and linoleic acid metabolism among upregulated genes. Therefore, the decrease in PC(18:0/20:4) may be related to selective turnover or remodeling of the AA-rich PC pool. Consistent with this, placental transcriptomic analysis showed enrichment of arachidonic acid and linoleic acid metabolism, together with upward changes in PLA2-family genes. Targeted RT-qPCR in an independent placental cohort further confirmed increased PLA2G4A and PLA2G2A expression, with a similar upward trend in PLA2G5. These findings provide transcription-level support for altered PLA2-related phospholipid turnover, although they do not directly demonstrate changes in enzyme activity or lipid flux.

The altered PC/PE balance may also involve changes in PC synthesis and maintenance. PC is mainly synthesized through the choline-dependent Kennedy pathway, but can also be generated from PE through phosphatidylethanolamine N-methyltransferase-mediated methylation. In an independent placental cohort, RT-qPCR showed that PCYT1A expression was significantly decreased, whereas PCYT2 showed a downward trend and CEPT1 and EPT1 remained unchanged. Reduced placental PCYT1A expression indicates altered transcriptional regulation of a key step in the CDP-choline pathway and provides complementary tissue-level context for the amniotic-fluid lipidomic findings. Together with the reduced PC abundance and PC/PE ratio observed in amniotic fluid, these independent findings converge on a preferential disturbance of PC homeostasis in PCOS-related pregnancies, rather than coordinated alteration of the Kennedy pathway as a whole.

The increase in PE(16:0/20:4) further suggests that AA may be redistributed into the PE pool rather than simply lost from membrane phospholipids. This pattern is consistent with deacylation–reacylation-mediated acyl-chain remodeling. However, placental RT-qPCR showed no significant differences in ACSL4, LPCAT3, or MBOAT7, providing limited transcription-level support for coordinated activation of Lands-cycle reacylation. Therefore, the decrease in AA-containing PC and the increase in AA-containing PE in amniotic fluid are unlikely to be driven by a single pathway. Instead, they may reflect the combined effects of selective phospholipid turnover, impaired maintenance of the PC pool, and redistribution of AA-containing acyl chains among phospholipid classes.

The lipid composition of amniotic fluid is jointly shaped by the fetus, fetal membranes, placenta, and maternal–fetal exchange. Membrane lipids such as PC, PE, and SM may be influenced by fetal lung secretions, as well as by membrane turnover and lipid release from placental, fetal membrane, or fetal tissues. The integration allows amniotic fluid to reflect the intrauterine metabolic environment directly encountered by the fetus, but also makes the precise tissue origin of individual differential lipids difficult to determine. Although maternal metabolic status may also contribute to amniotic-fluid lipid composition, the identified phospholipid alterations were unlikely to be primarily explained by differences in maternal adiposity, as these alterations persisted after considering maternal pre-pregnancy BMI. Thus, the placental transcriptomic and RT-qPCR findings provide complementary tissue-level evidence for related phospholipid pathways, although they do not establish the principal tissue source of the altered amniotic-fluid lipids.

A similar direction of lipid alteration has also been reported in animal models of gestational hyperandrogen exposure (45). In a sheep model of testosterone excess during early to mid-gestation, Saadat et al. found that hyperandrogen exposure was associated not only with fetal growth restriction and cardiometabolic abnormalities in the offspring, but also with decreased maternal plasma PC and key unsaturated fatty acids, together with alterations in PE and PI biosynthetic pathways. These findings suggest that androgen excess during pregnancy may be accompanied by a shift in glycerophospholipid metabolism toward lower PC abundance and altered PE-related biosynthesis. The present study further identified similar PC/PE-related abnormalities in amniotic fluid, which represents the immediate prenatal exposure environment of the fetus. This suggests that such membrane lipid alterations may not be limited to the maternal circulation, but may already be present in the intrauterine fluid environment surrounding the fetus.

The potential relationship between this lipid metabolic pattern and fetal development is also supported by previous studies and by the birth-weight analysis in the present study. Metabolomic studies of PCOS-related pregnancies have reported lower offspring birth weight together with a metabolic signature enriched in lipids containing arachidonic acid, linoleic acid, and palmitic acid (46). Plasma lipidomic profiling of children born to mothers with PCOS has also shown abnormalities in glycerophospholipids and sphingomyelins, with several PC, LPC, and SM species associated with glucose metabolism and cardiovascular risk markers (47). Amniotic fluid is not a simple reflection of maternal circulating lipids, but a local environment jointly shaped by the fetus, fetal membranes, and the fetal side of the placenta (38). Recent amniotic fluid lipidomic studies have further shown that alterations in amniotic fluid lipids may be associated with fetal growth phenotypes (48). In the present study, PE(16:0/20:4) remained negatively associated with birth weight after multivariable adjustment, and this association was stable across several sensitivity analyses. These findings suggest that enrichment of AA-containing PE may represent a metabolic link between phospholipid remodeling in amniotic fluid and fetal growth status. Although the present results do not establish causality, they show that PCOS-related lipid disturbances, prenatal fetal exposure, and neonatal growth phenotypes can be observed within the same amniotic fluid metabolic window. They also provide a basis for further investigation of the potential links between the PCOS-related intrauterine environment and the long-term cardiometabolic and reproductive susceptibility of the offspring (14, 49, 50).

Abnormalities in polyunsaturated fatty acid metabolism provide an additional metabolic context for the membrane phospholipid remodeling described above. Polyunsaturated fatty acids (PUFAs) are fatty acids containing two or more double bonds. They can serve as acyl-chain components of membrane phospholipids such as PC and PE, and can also act as precursors of various bioactive lipid mediators (51). In the present study, α-linolenic acid metabolism showed a relatively high pathway impact value and statistical significance, whereas linoleic acid metabolism and arachidonic acid metabolism were not enriched to the same extent. This pattern suggests that the fatty acid abnormalities in PCOS amniotic fluid do not represent a broad disturbance of fatty acid metabolism. Rather, they may reflect changes in specific PUFA-related pathways. α-Linolenic acid is an important initiating fatty acid in n-3 PUFA metabolism and serves as a metabolic precursor of EPA and DHA. It has also been reported to participate in metabolic programming during fetal development (52). Meanwhile, n-3 and n-6 PUFA pathways together influence membrane phospholipid acyl-chain composition and lipid mediator production. Therefore, altered α-linolenic acid metabolism may provide a metabolic background for the changes in AA-containing PC and PE species observed in this study. It may also suggest a possible shift in the n-3/n-6 PUFA balance within the fetal intrauterine environment. This interpretation is partly consistent with previous metabolomic studies of PCOS-related pregnancies. Diboun et al (46). reported that PCOS-related pregnancies were associated with lower birth weight and showed a metabolic signature enriched in specific triglycerides containing arachidonic acid, linoleic acid, and palmitic acid, as well as unsaturated fatty acids. Another metabolomic study (53) of placenta and umbilical cord serum also showed alterations in unsaturated fatty acid-related metabolites at the maternal–fetal interface in PCOS-related pregnancies, including adrenic acid and γ-linolenic acid. Together with the changes in AA-containing PC and PE species observed in the present study, the enrichment of arachidonic acid and linoleic acid metabolism among upregulated placental genes provides additional support for PUFA-related metabolic alterations in PCOS-related pregnancies. Thus, PUFA-related metabolic alterations may not constitute the central mechanism of the present study, but they provide a supportive link between membrane phospholipid acyl-chain remodeling, lipid-signaling alterations, and fetal growth outcomes. Given that changes in the PC/PE balance and AA-containing phospholipid species were more direct and consistent in our data, α-linolenic acid metabolism should still be interpreted as an auxiliary lipid metabolic signal, rather than a core driver of amniotic-fluid metabolic remodeling in PCOS-related pregnancies.

Beyond membrane phospholipid remodeling, abnormalities in sphingolipid metabolism further suggest that lipid alterations in PCOS amniotic fluid may extend from membrane structural remodeling to lipid-mediated signaling regulation. In the present study, the sphingolipid signaling pathway emerged as an important enriched node in KEGG analysis. Sphingolipid metabolism was also significantly enriched in the lipid metabolism module. Further class-enrichment analysis showed that sphingolipid metabolites were overrepresented among the core metabolites, with SM(d18:2(4E,14Z)/16:0) showing a clear increase in the PCOS group. These results suggest that sphingolipid alterations in PCOS amniotic fluid are unlikely to be random findings. Rather, they may represent an important component of the broader lipid remodeling network. Sphingolipids are not only important components of cell membranes and lipid rafts, but also bioactive lipid molecules involved in signal regulation. Previous sphingolipidomic profiling has reported increased levels of S1P, ceramide, and sphingomyelin in the serum of women with PCOS, supporting a potential link between sphingolipid dysregulation and insulin resistance, inflammation, and metabolic disturbance in PCOS (54). In pregnancy, S1P and ceramide are involved in decidualization, trophoblast invasion, placental vascularization, and placental barrier development (55). Abnormal levels of these sphingolipids have also been associated with pregnancy complications, including preeclampsia, gestational diabetes mellitus, and fetal growth restriction (56). Therefore, the enrichment of sphingolipid metabolism observed in PCOS amniotic fluid suggests that lipid abnormalities in the fetal intrauterine environment may not be limited to changes in membrane phospholipid composition, but may also involve metabolic signaling at the placenta–fetus interface. This observation is in line with previous offspring lipidomic evidence (57) and supports the possibility that sphingolipid-related alterations may represent an early signal of later metabolic susceptibility. In this context, the sphingolipid abnormalities observed in amniotic fluid may represent an earlier intrauterine lipid exposure signal, consistent with offspring lipid-metabolic susceptibility in PCOS-related pregnancies.

In addition to the changes in lipid metabolism, a small number of steroid-related metabolites were increased in the amniotic fluid of the PCOS group, mainly including direct and indirect androgen derivatives and estrogen-related metabolites. This pattern is broadly consistent with the hyperandrogenic background of PCOS (58, 59). The increase in estrogen-related metabolites may be associated with the aromatization of androgen precursors or other enzymatic conversion pathways. However, the steroid composition of amniotic fluid is influenced by maternal, placental, and fetal metabolism. These findings should therefore be interpreted as changes in selected steroid metabolites and are not sufficient to indicate a generalized alteration of the fetal endocrine environment.

Multilevel sensitivity analyses further demonstrated that the core metabolites remained highly consistent under different analytical conditions. Given the higher proportions of assisted reproduction, twin pregnancy, and pregnancy complications in the PCOS group, these analyses are particularly important for evaluating the robustness of the findings. The persistence of the core metabolic signature after excluding these potential confounding factors suggests that the major lipid features and the changes in a small number of steroid-related metabolites are unlikely to be explained entirely by clinical heterogeneity. Instead, they may represent relatively stable metabolic features of the intrauterine environment in PCOS-related pregnancies.

Placental transcriptomic analysis revealed a clear immune-related signature. Enriched pathways included antigen processing and presentation, MHC class II-related processes, cytokine–cytokine receptor interaction, natural killer cell-mediated cytotoxicity, and complement and coagulation pathways. Similar immune alterations have been reported in placental tissue from women with PCOS (60), including changes in immune-cell infiltration and immune-related regulatory networks, suggesting that immune dysregulation may persist at the maternal–fetal interface during late gestation. These findings should, however, be interpreted cautiously because bulk placental RNA-seq cannot distinguish changes in immune-cell composition from transcriptional alterations within placental cells. The observed enrichment therefore supports an immune-related placental signature rather than a defined immune mechanism. Notably, arachidonic acid and linoleic acid metabolism were also enriched in the same dataset. As membrane phospholipids are a major source of arachidonic acid and related bioactive lipid mediators, the parallel changes in lipid- and immune-related pathways may reflect a broader disturbance of the placental metabolic–immune environment and provide additional context for the phospholipid abnormalities observed in amniotic fluid (61).

Overall, the metabolic alterations in the amniotic fluid of PCOS-related pregnancies were characterized primarily by disturbances in membrane lipid homeostasis. The most evident changes were the altered PC/PE balance and the redistribution of AA-containing phospholipid species, accompanied by changes in selected PUFA pathways, sphingolipid metabolism, and a small number of steroid-related metabolites. The placental transcriptomic and RT-qPCR findings provided corresponding evidence for some of these phospholipid changes. The consistent negative association between PE(16:0/20:4) and birth weight further suggests that the altered lipid profile may be related to fetal growth. These findings indicate that amniotic fluid captures metabolic disturbances associated with PCOS pregnancy and, importantly, reflects the intrauterine lipid environment to which the fetus is directly exposed.

However, this study still has certain limitations. Firstly, the sample size was relatively small, and the PCOS group showed a certain degree of clinical heterogeneity, including higher proportions of assisted reproduction, twin pregnancy, and pregnancy complications. Although multivariable adjustment and sensitivity analyses supported the stability of the main findings, residual confounding cannot be completely excluded. In addition, direct measures of maternal insulin sensitivity were not systematically available in the present cohort; therefore, the potential contribution of maternal insulin resistance to the observed lipid alterations cannot be fully excluded. Secondly, paired maternal blood, placental tissue, cord blood, and amniotic fluid samples were not available. Therefore, the main sources of the altered metabolites could not be determined, and it remains unclear whether these changes reflect consequences of the altered intrauterine environment, compensatory responses, or processes involved in fetal growth regulation. In addition, the placental RNA-seq cohort was small, and differential expression was based on a nominal P-value threshold. The independent RT-qPCR cohort was also limited in size. Therefore, the placental molecular findings should be regarded as exploratory pilot evidence that provides tissue-level context for the amniotic-fluid metabolic signature rather than definitive mechanistic or confirmatory evidence. The observed pathway-level concordance, particularly for PLA2-related phospholipid turnover, requires replication in larger, well-characterized placental cohorts. Finally, targeted lipid validation and long-term offspring follow-up were not performed. Future studies incorporating paired maternal–fetal samples, targeted lipid measurements, and offspring follow-up are needed to further clarify the biological basis and long-term relevance of these findings.

## 5 Conclusions

This study conducted a non-targeted metabolomic analysis of amniotic fluid from women with PCOS during late pregnancy and revealed broad metabolic abnormalities within the intrauterine environment. These alterations were dominated by lipid metabolic remodeling, particularly membrane phospholipid remodeling, PC/PE imbalance, and redistribution of AA-containing phospholipid species, with additional involvement of sphingolipid- and PUFA-related pathways. Steroid hormone-related metabolites were also altered, providing supporting evidence for an abnormal endocrine exposure environment in PCOS-related pregnancies. Multilevel sensitivity analyses showed that these metabolic signatures were largely retained after accounting for clinical heterogeneity, supporting their robustness in PCOS-related pregnancies.

Integration of amniotic fluid metabolomics with exploratory placental transcriptomic analysis and targeted RT-qPCR assessment in an independent sample set provided complementary evidence for phospholipid-related alterations at the maternal–fetal interface and suggested cross-cohort convergence at the pathway level. Placental transcriptomic analysis also identified accompanying immune-related alterations, although their cellular basis and functional significance require further investigation. Together, these findings indicate that PCOS-related pregnancies are characterized by altered amniotic-fluid lipid metabolism together with selected placental molecular changes, providing a basis for further studies of intrauterine metabolic exposure, fetal growth, and long-term metabolic risk in the offspring.

## Data Availability

The datasets presented in this study can be found in online repositories. The names of the repository/repositories and accession number(s) can be found in the article/**Supplementary Material**.
