# Supplementary material for: Amniotic-fluid metabolomics identifies phospholipid remodeling as a metabolic signature of intrauterine exposure in pregnancies with polycystic ovary syndrome
Source: Front Endocrinol (Lausanne). 2026 Sep 4;17:1904695. doi: 10.3389/fendo.2026.1904695 (PMC13585535; doi:10.3389/fendo.2026.1904695)
Supplement: Supplementary file 1 [file DataSheet1.docx]

**Supplementary Figure S1. Principal component analysis (PCA) score plot of amniotic fluid metabolomic profiles.**

PCA was performed on normalized metabolite data from all samples, including control, PCOS, and quality control (QC) samples. Each point represents an individual sample. QC samples are tightly clustered, indicating good analytical stability and reproducibility of the metabolomic data. The first two principal components (PC1 and PC2) explain 12.38% and 8.38% of the total variance, respectively.

**Supplementary Figure S2. Pearson correlation analysis of quality control (QC) samples.**

Pairwise Pearson correlation analysis was performed among QC samples using normalized metabolite data. The correlation coefficients (r) were consistently high (r > 0.9), with most values close to 1.0, demonstrating excellent reproducibility and reliability of the analytical platform.Pearson correlation analysis of the quality control samples yielded values above 0.9, indicating good correlation.

**Supplementary Figure S3. Hotelling’s T² statistic for outlier detection in metabolomic data.**

Hotelling’s T² analysis was performed to assess potential outliers in the dataset based on multivariate modeling. The dashed horizontal lines indicate the 95% and 99% confidence limits. Each point represents an individual sample, and samples are color-coded according to group (control, PCOS, and QC). No samples exceeded the critical thresholds, suggesting the absence of strong outliers and supporting the robustness of the dataset.

**Supplementary Figure S4. Orthogonal partial least squares discriminant analysis (OPLS–DA) score plot of amniotic fluid metabolomic profiles.**

To differentiate between the control and PCOS groups, an OPLS–DA model was built using normalized metabolite data. An individual sample is represented by each point. Significant metabolic differences were shown by the two groups' good separation. R²X, R²Y, and Q² parameters were used to assess the model's performance; Q² denotes predictive ability while R² represents the explained variance. With Q2 > 0.5, the model demonstrated strong stability and predictive power.

**Supplementary Figure S5. KEGG pathway enrichment analysis of differential metabolites.** Pathway Impact (network topology) is shown on the x-axis. Bubble size reflects the number of differential metabolites mapped to each route (count), whereas color indicates fold enrichment. Glycerophospholipid metabolism and the sphingolipid signaling pathway stood out for their strong impact and statistical significance.

**Supplementary Figure S6. Consistency scatter plots of log2FC values between the full cohort and each sensitivity analysis.** (A) Full cohort vs. excluding complications; (B) full cohort vs. excluding twins; (C) full cohort vs. excluding IVF. Each point represents one metabolite (n = 89). The dashed line denotes y = x, and the shaded area indicates the 95% confidence interval of the linear fit. Circles, upregulated metabolites; diamonds, downregulated metabolites; squares, non-significant metabolites. Strong concordance was observed in all comparisons, with r = 0.985 and R² = 0.971 after excluding complications, r = 0.996 and R² = 0.992 after excluding twins, and r = 0.999 and R² = 0.998 after excluding IVF (P < 2.2 × 10⁻¹⁶). Metabolites clustered closely around the diagonal, with no reversal in direction of change, indicating that these clinical factors caused only minor variation in absolute log2FC values without altering the overall pattern of differential expression.

**Supplementary Figure S7. UpSet intersection plot of significant differential metabolites across four sensitivity analyses.**

The upper bar chart indicates the size of each intersection, and the connected dots in the lower matrix denote the corresponding analysis combinations. In total, 347 metabolites were significant in at least one analysis. Of these, 135 (38.9%) were shared across all four analyses, representing the largest intersection set. The complication-excluded analysis contributed the most unique metabolites, with 68 metabolites unique to this analysis (19.6%), whereas the full cohort contributed only 8 unique metabolites. In addition, 34 metabolites were specifically shared between the complication-excluded and twin-excluded analyses. After additional VIP and FDR filtering, 89 metabolites were retained as the final core set.

**Supplementary Figure S8. Stacked bar chart of class distribution of significant differential metabolites across four sensitivity analyses.** Stacked bar chart showing the chemical class composition of significant differential metabolites (DEMs) in the full cohort and in the analyses excluding pregnancies with complications, twin gestations, or IVF conception. Numbers within bars indicate metabolite counts per class, and totals are shown above bars. In the full cohort, 193 DEMs were identified, of which fatty acid derivatives (39, 20.2%) and phospholipids (29, 15.0%) were the dominant classes; lysophospholipids (7, 3.6%), sphingolipids (6, 3.1%), glycerolipids (4, 2.1%), and triglycerides (2, 1.0%) were present in smaller numbers, whereas 106 metabolites (54.9%) belonged to other classes. Excluding pregnancies with complications increased the total number of DEMs to 274, driven mainly by fatty acid derivatives (39 to 65) and lysophospholipids (7 to 15), while phospholipids remained unchanged (29 vs. 29). The twin-excluded and IVF-excluded analyses identified 232 and 210 DEMs, respectively, and showed broadly similar class distributions. Lipid-related classes accounted for approximately 40–50% of DEMs in all four analyses.

**Supplementary Figure S9. Sample-to-sample Pearson correlation heatmap of placental transcriptomic profiles.**

Pearson correlation coefficients were calculated using normalized gene expression values across placental RNA-seq samples. Most samples showed high overall transcriptomic similarity, although inter-individual variability was present. Given the small sample size, the dataset was used for targeted tissue-level interpretation rather than broad transcriptome-wide discovery.

**Supplementary Figure S10.** **Associations between PCOS status and representative amniotic fluid lipid features before and after adjustment for pre-pregnancy BMI.**

Ordinary least-squares regression models were fitted for 12 representative metabolites and the phosphatidylcholine/phosphatidylethanolamine (PC/PE) ratio, with metabolite abundance as the dependent variable and PCOS status as the independent variable, before and after adjustment for pre-pregnancy BMI (n = 45). Pre-pregnancy BMI did not differ between control and PCOS groups (22.35 ± 3.38 vs 23.78 ± 3.89 kg/m²; Welch t = 1.32, P = 0.194). None of the 13 lipid indices correlated significantly with pre-pregnancy BMI (Spearman, all P ≥ 0.102). Points represent standardized regression coefficients (β), and horizontal bars indicate 95% confidence intervals.
